# Human Immune Cell Epigenomic Signatures in Response to Infectious Diseases and Chemical Exposures

**DOI:** 10.1101/2023.06.29.546792

**Published:** 2023-06-30

**Authors:** Wenliang Wang, Manoj Hariharan, Anna Bartlett, Cesar Barragan, Rosa Castanon, Vince Rothenberg, Haili Song, Joseph Nery, Andrew Aldridge, Jordan Altshul, Mia Kenworthy, Wubin Ding, Hanqing Liu, Wei Tian, Jingtian Zhou, Huaming Chen, Bei Wei, Irem B. Gündüz, Todd Norell, Timothy J Broderick, Micah T. McClain, Lisa L. Satterwhite, Thomas W. Burke, Elizabeth A. Petzold, Xiling Shen, Christopher W. Woods, Vance G. Fowler, Felicia Ruffin, Parinya Panuwet, Dana B. Barr, Jennifer L. Beare, Anthony K. Smith, Rachel R. Spurbeck, Sindhu Vangeti, Irene Ramos, German Nudelman, Stuart C. Sealfon, Flora Castellino, Anna Maria Walley, Thomas Evans, Fabian Müller, William J. Greenleaf, Joseph R. Ecker

**Affiliations:** 1 Genomic Analysis Laboratory, The Salk Institute for Biological Studies, 10010 N Torrey Pines Rd, La Jolla, CA 92037, USA; 2 Duke University School of Medicine, Bryan Research Building, 311 Research Drive, Durham, NC 27710, USA; 3 Department of Genetics, Stanford University, Stanford, CA 94305, USA; 4 Integrative Cellular Biology & Bioinformatics Lab, Saarland University, 66123 Saarbrücken, Germany; 5 Healthspan, Resilience, and Performance, Florida Institute for Human and Machine Cognition, 40 S Alcaniz St, Pensacola, FL 32502, USA; 6 Center for Infectious Disease Diagnostics and Innovation, Division of Infectious Diseases, Duke University Medical Center, Durham, NC 27710 USA; 7 Durham Veterans Affairs Medical Center, Durham, NC 27705 USA; 8 Department of Civil and Environmental Engineering, Pratt School of Engineering, Duke University, Durham, NC 27708, USA; 9 Terasaki Institute for Biomedical Innovation, Los Angeles, CA 90024, USA; 10 Duke Clinical Research Institute, Durham NC 27701 USA; 11 Gangarosa Department of Environmental Health, Rollins School of Public Health, Emory University, Atlanta, GA 30322 USA; 12 Battelle Memorial Institute, 505 King Ave Columbus OH 43201, USA; 13 Department of Neurology, Icahn School of Medicine at Mount Sinai; New York, NY 10029, USA; 14 U.S. Department of Health and Human Services, Administration for Strategic Preparedness and Response, Biomedical Advanced Research and Development Authority, Washington, DC, USA.; 15 Vaccitech plc, Unit 6-10, Zeus Building, Rutherford Avenue, Harwell OX11 0DF, United Kingdom; 16 Howard Hughes Medical Institute, The Salk Institute for Biological Studies, 10010 N Torrey Pines Rd, La Jolla, CA 92037, USA

**Keywords:** Epigenome, DNA methylation, Exposure, Infection, Immune cells

## Abstract

Variations in DNA methylation patterns in human tissues have been linked to various environmental exposures and infections. Here, we identified the DNA methylation signatures associated with multiple exposures in nine major immune cell types derived from peripheral blood mononuclear cells (PBMCs) at single-cell resolution. We performed methylome sequencing on 111,180 immune cells obtained from 112 individuals who were exposed to different viruses, bacteria, or chemicals. Our analysis revealed 790,662 differentially methylated regions (DMRs) associated with these exposures, which are mostly individual CpG sites. Additionally, we integrated methylation and ATAC-seq data from same samples and found strong correlations between the two modalities. However, the epigenomic remodeling in these two modalities are complementary. Finally, we identified the minimum set of DMRs that can predict exposures. Overall, our study provides the first comprehensive dataset of single immune cell methylation profiles, along with unique methylation biomarkers for various biological and chemical exposures.

## Introduction

Infectious diseases are among the greatest threats to human and animal health worldwide and are among the top ten causes of death (Bloom and Cadarette 2019). The Severe Acute Respiratory Syndrome Coronavirus 2 (SARS-CoV-2) global pandemic that began in early 2020 is still a major public health issue and has caused millions of deaths and dramatically impacted economies worldwide (Sachs et al. 2022). Human Immunodeficiency Virus type 1 (HIV-1) is a lentivirus that causes Acquired Immunodeficiency Syndrome (AIDS), a condition in which the immune system fails to defend the host, increasing susceptibility to opportunistic infectious diseases and malignancies (Powell et al. 2016; McLaren and Fellay 2021). Since the first report in 1981, HIV has been a major public health issue for over four decades and has claimed more than 40 million lives, according to the Joint United Nations Programme on HIV/AIDS (UNAIDS) (Unaids and UNAIDS 2022). Influenza is an acute respiratory infection caused by the influenza viruses, which circulate in all parts of the world and infects 5%–15% of the population in the United States each year (Tokars, Olsen, and Reed 2018). Besides viral infections, bacterial infections are also a significant threat to public health. Methicillin-resistant *Staphylococcus aureus* (MRSA) is a common bacteria resistant to multiple drugs and antibiotics. It is reported by the United States Centers for Disease Control and Prevention (CDC) that approximately 5% of patients in U.S. hospitals develop nosocomial MRSA infection (Nandhini et al. 2022). In contrast, methicillin-susceptible Staphylococcus aureus (MSSA), is sensitive to methicillin and other antibiotics. Both MRSA and MSSA can cause life-threatening infections. Anthrax infection has been known in cattle and mankind for several centuries, caused mainly by the Bacillus anthracis bacteria which harbors both pXO1 and pXO2 virulence plasmids. While naturally occurring human infections are less common compared to outbreaks in animals, this pathogen is notorious for its potential as a bioweapon (Swartz 2001). The toxin can inhibit the immune responses and lead to severe respiratory illness, pneumonia, and even death (Bower et al. 2022). Chemical exposures also pose ongoing threats to human health, such as organophosphate pesticides, which are associated with neurodevelopmental and congenital disabilities (Hertz-Picciotto et al. 2018). Furthermore, exposure to organophosphate esters can reduce the immune response to childhood vaccines in children (Hammel et al. 2022).

Cytosine DNA methylation plays a crucial role in regulating immune cell lineage and the host’s response to pathogens (Calle-Fabregat, Morante-Palacios, and Ballestar 2020; Roy et al. 2021). During the development of immune cells from hematopoietic stem cells (HSCs), both the global methylation level and lineage-specific methylation undergo changes that are influenced by lineage-specific transcription factors (Ji et al. 2010; Calle-Fabregat, Morante-Palacios, and Ballestar 2020; Roy et al. 2021). Although DNA methylation was previously considered relatively stable compared to other epigenetic modifications, recent studies have shown that it can be altered immediately after pathogen infection or exposure to environmental factors (Pacis et al., n.d.; Chang et al. 2021; Martin and Fry 2018). These changes in CpG sites have the potential to serve as biomarkers for both acute and chronic infectious disease diagnosis.

To investigate the DNA methylation alterations during the immune response to pathogens and toxic chemicals in major innate and adaptive immune cell types, we isolated seven cell types (B cells, Monocytes, NK cells, CD8 memory T cells, CD8 naïve T cells, CD4 memory T cells, and CD4 naïve T cells) from PBMCs of patients and healthy controls, and performed single-nucleus methylation sequencing (snmC-seq2) (Luo et al. 2017, 2018). We characterized the cell types based on fluorescence-activated cell sorting (FACS) and genome-wide methylation profiles in the CG context of each cell. This analysis revealed two sub-clusters within B cells and NK cells. We then identified differentially methylated regions (DMRs) within each of these nine cell types. For the cohorts exposed to viruses, bacteria, and chemicals, we conducted snmC-seq2 on the seven cell types using 173 PBMC samples collected from 112 donors, resulting in 111,180 PBMC methylation profiles. We examined the impact of each exposure on the methylome of the immune cell types and trained a model using these DMRs to predict different types of exposures based on the methylation status of several thousand loci. Additionally, we generated single-nucleus ATAC-seq data from patients with the same exposures and found a high correlation between these two modalities. However, we observed minimal overlap between the exposure-associated changes in methylation and chromatin accessibility, indicating the need to study epigenomic remodeling associated with exposures using both techniques.

Our study provides valuable resources and insights into the molecular mechanisms underlying the impact of different exposures on DNA methylation in immune cells. To our knowledge, this is the first large-scale, single-cell DNA methylation dataset on major immune cell types, which will be instrumental in developing diagnostic assays for detecting and containing infections at their source. Moreover, the potential gene regulatory effects of differential methylation in intergenic regions offer new insights into human pathogenesis. The unique DNA methylation signatures identified in this study contribute to our understanding of the epigenetic regulation of immune processes and offer a novel approach to diagnosing exposures to known and unknown pathogens.

## Results

**Figure 1.**
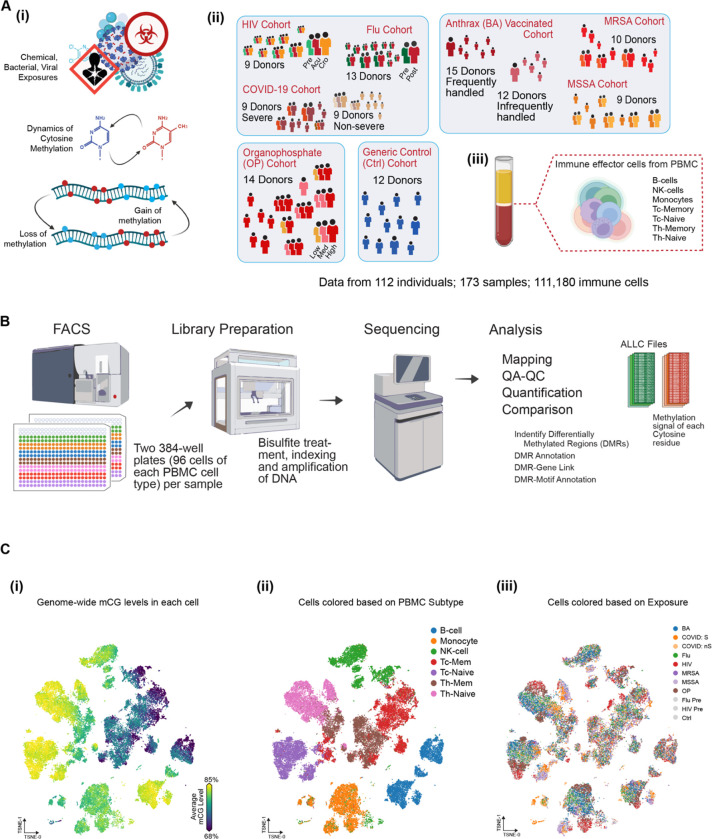
Single-nucleus methylation sequencing of PBMCs from individuals with different exposures. **A**. Samples from cohorts with viral, bacterial, or chemical exposures. **B**. Experimental and analysis pipelines. PBMCs were collected from individuals with different exposures, sorted into seven immune cell types on 384-well plates, and subjected to single-nucleus methylation sequencing. Data analysis was performed using our pipeline. **C.** Single nucleus clustering of cells from all exposures, colored by cell surface markers (i), global mCG levels (ii), and different exposures (iii).

In this study, our main focus was on three categories of exposures: viral, bacterial, and chemical (Figure 1A). To examine viral exposure, we evaluated the methylation patterns of individuals who were part of a prospective study on HIV-1 infection prevention. Additionally, we analyzed a cohort of volunteers who participated in a vaccine trial against the H3N2 flu virus and a group of patients who experienced SARS-CoV-2 infection with varying degrees of disease severity. For bacterial exposures, we examined the methylation profiles of patients infected with MRSA and MSSA, as well as vaccinated technicians who handled Bacillus anthracis. In terms of chemical exposure, we obtained samples from individuals with high levels of 3,5,6-trichloro-2-pyridinol (TCPY) resulting from exposure to chlorpyrifos—an organophosphate insecticide known to be neurotoxic and shares a mode of action with nerve agents. Detailed information about each of these cohorts can be found in the Subject Details section, and sample metadata are provided in Table S1. Peripheral blood mononuclear cells (PBMCs) were collected from each of these cohorts, and we isolated and sorted seven major immune cell types (Figure 1B, S1). To account for potential batch effects, we sorted all seven immune cell types on the same 384-well plate. Subsequently, we profiled the methylomes of each single cell using snmC-seq2 (Luo et al., 2017, 2018) and processed the data using ALLCools (Liu et al., 2021)—a custom bioinformatic pipeline for analyzing single-nucleus methylation data. We then performed Leiden clustering and t-distributed stochastic neighbor embedding (t-SNE) analysis of the single nuclei based on their methylation profiles across all cells (Figure 1C). While immune cell types were initially sorted using cell surface markers, the global mCG levels enabled us to identify unique sub-clusters within B cells and NK cells (Figure 1C). Importantly, cells from different exposures were not evenly distributed across these sub-clusters, indicating the presence of distinct epigenomic diversity associated with exposure types (Figure 1C).

### Methylation Signatures in Immune Cells from Healthy Donors at Single-Cell Resolution

**Figure 2.**
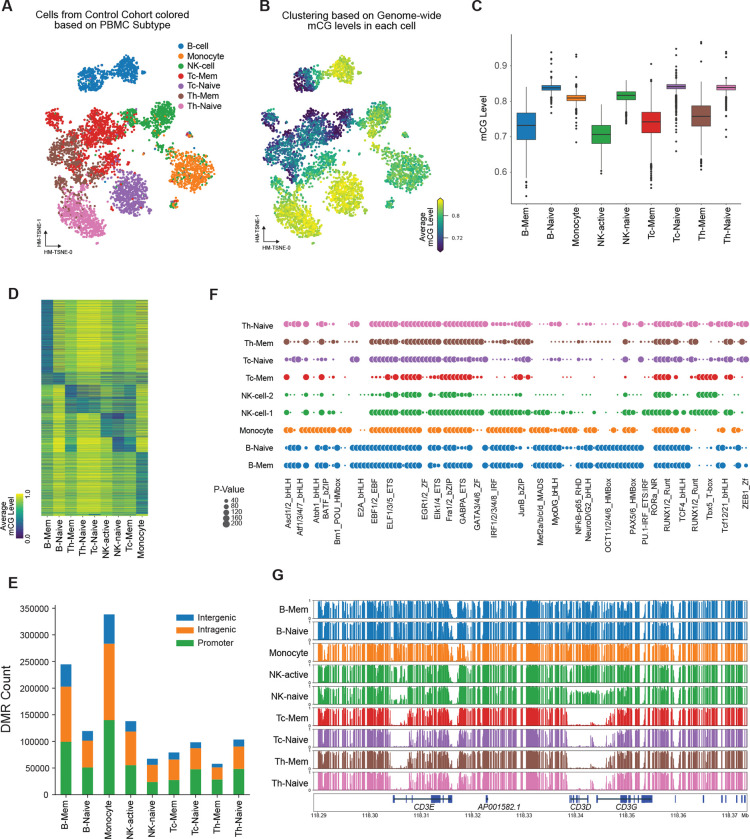
Cell type-specific methylation signatures of immune cells from healthy controls. **A**. t-distributed stochastic neighbor embedding (t-SNE) embeddings of single-nucleus methylomes from healthy controls, colored by cell surface markers. **B.** Same embedding of cells colored by global mCG levels. **C.** Global mCG level of different cell types defined by cell surface markers and single-nucleus methylome. **D.** Heatmap showing the mCG methylation level at cell type-specific DMRs. **E.** Bar plot showing the count of DMRs located in intergenic, intragenic, and promoter regions for each cell type. **F**. Scatter plot showing the enrichment of motifs at DMRs for each cell type. The size of the dot indicates the enrichment p-value, while the colors represent the cell types. **G.** Genome browser view of DNA methylation levels at T cell marker genes CD3D, CD3E, and CD3G, which are hypomethylated in the T cell lineage and hypermethylated in other cell types.

To elucidate the cell type-specific methylation signatures for major immune cell classes at single-cell resolution, we obtained peripheral blood mononuclear cells (PBMCs) from healthy donors across different cohorts. As outlined in our methodology (Figure 1), we performed fluorescence-activated cell sorting (FACS) to isolate seven major immune cell types from PBMCs, followed by single-cell methylation sequencing (Luo et al., 2017, 2018). Methylation reads from individual immune cells covered approximately 5%–10% of the genome (Figure S2A) and underwent filtering based on read mapping rate, total read count, and global CpG methylation (mCG) level (Figure S2B–D). In total, we obtained 22,341 high-quality cells from healthy donors that passed the filtering step and were used for subsequent analysis. To cluster these single cells, we partitioned the genome into 5 kb bins, generating a cell-by-bin matrix (Liu et al., 2021), upon which clustering and t-SNE were performed. After incorporating Harmony (Korsunsky et al., 2019) to account for donor effects, our results demonstrated well-mixed cells from different cohorts (Figure S2E), affirming the reliability of our clustering outcomes. Cell type assignment was further refined based on cell surface markers and single-nucleus methylation clustering (Figure 2A, S2F). Additionally, by considering the global mCG levels of single nuclei, we identified two subpopulations within B cells and NK cells, denoted as memory B cells (B-Mem), naive B cells (B-Naive), activated NK cells (NK-Active), and naive NK cells (NK-Naive) (Figure 2A–C), respectively. Interestingly, CD4 and CD8 naive T-cells exhibited higher global methylation levels and formed distinct clusters, while CD4 and CD8 memory T cells displayed more diverse methylation profiles and lower global mCG levels (Figure 2A–C).

To identify potential regulatory elements in different immune cells, we detected hypomethylated differentially methylated regions (DMRs) for each cell type. Given the considerable variation in global mCG levels among these clusters (Figure 2C), we performed separate DMR calling between cell types with low mCG and high mCG. In total, 495,479 DMRs were identified across the immune cell types, with most being unique to a specific cell type (Figure 2D). Notably, subclusters of B cells and T cells shared only a small number of DMRs (Figure S2G), while NK cells exhibited more DMRs shared with Tc-Mem cells than with other T cell subtypes (Figure 2D, S2G). To gain insights into their potential impact on gene regulation, we associated each DMR with underlying genomic features and observed significant enrichment in intragenic and promoter regions for each immune cell type (Figure 2E). Motif analysis using DMRs revealed the enrichment of known lineage-specific transcription factor binding sites in corresponding cell types (Figure 2F). For instance, TCF motifs were enriched in T cells, while the EBF1 motif showed enrichment in B cells (Figure 2F). To further validate the accuracy of our data, we plotted the methylation status at T cell marker CD3 genes, which revealed hypomethylation in T cells and hypermethylation in other cell types (Figure 2G).

### Impact of HIV-1 Infection on the Methylome

**Figure 3.**
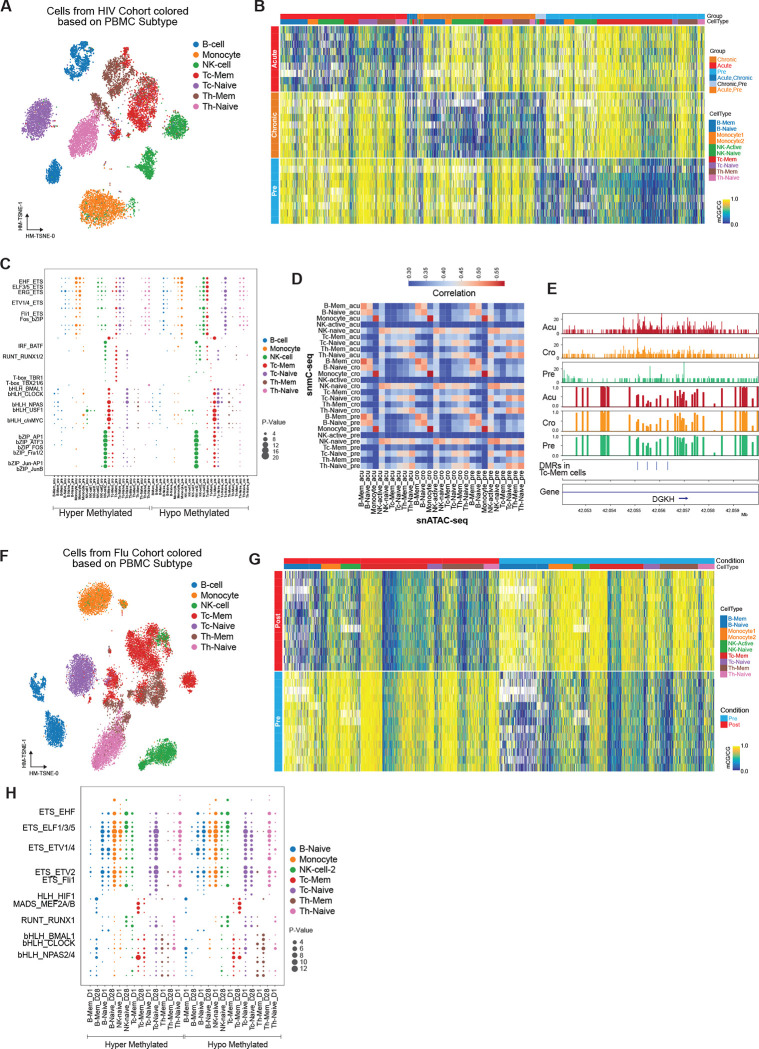
Impact of HIV-1 and influenza infection on methylomes of immune cells. **A**. t-SNE embeddings of single-nucleus methylomes from the HIV-1 cohort, representing ‘pre,’ ‘acute,’ and ‘chronic’ stages. Color indicates the cell surface markers of these cells. **B**. Heatmap showing the mCG level at DMRs between ‘pre,’ ‘acute,’ and ‘chronic’ samples. The color scale represents methylation levels. DMRs are grouped by hypomethylation and further grouped by cell types. **C.** Scatter plot showing enriched motifs of DMRs between the three conditions (‘pre,’ ‘acute,’ and ‘chronic’) in all cell types. The color represents cell types defined by cell surface markers, and the size of the dots indicates the enrichment p-value. **D**. Heatmap showing genome-wide correlations between pseudo-bulk signals from each cell type in single-nucleus methylation and matched single-nucleus ATAC-seq. Red indicates stronger correlation, while blue indicates weaker correlation. **E.** Genome browser view of pseudo-bulk single-nucleus ATAC and methylation signals from memory CD8 T cells at the intron of gene DGKH, showing signals from three stages of HIV-1 infection, with colors indicating different stages. **F.** t-SNE embeddings of single-nucleus methylomes from the influenza cohort, representing ‘pre’ and ‘post’ stages. Color indicates the cell surface markers of these cells. **G.** Heatmap showing the mCG level at DMRs between ‘pre’ and ‘post’ samples. The color scale represents methylation levels. DMRs are grouped by hypomethylation and further grouped by cell types. **H.** Scatter plot showing enriched motifs of DMRs between the two conditions (‘pre’ and ‘post’) in all cell types. The color represents cell types defined by cell surface markers, and the size of the dots indicates the enrichment p-value.

To investigate the impact of HIV-1 infection on the methylome of immune cells, we conducted a comprehensive analysis using immune cells obtained from the same individuals at different stages: “pre” (before infection), “acute” (after diagnosis), and “chronic” (after treatment) (Figure 1). We employed snmC-seq2 (Luo et al., 2017, 2018) to profile the methylome of these cells. Based on the methylation profiles, we identified 13 clusters of immune cells (Figure S3A) and annotated them based on both cell surface markers and methylation profiles. These cell clusters were evenly distributed across the three stages of the disease (Figure S3B). Two sub-clusters of B cells and NK cells were identified based on methylation and cell surface markers, while other cell types were assigned based on cell surface markers (Figure 3A). Similar to the analysis performed on healthy control samples, we classified two clusters of B cells as B-Mem and B-Naive, and two NK cell clusters as NK-Active and NK-Naive, based on the global mCG level (Figure S3C). The ratio between B-Mem and B-Naive remained consistent across the three stages of HIV-1 infection. However, the ratio between NK cell sub-clusters varied between the “acute,” “chronic,” and “pre” stages, with “pre” samples exhibiting fewer NK-Active cells and more NK-Naive cells (Figure S3D) (P=0.0073, Chi-square test, comparing “acute” and “pre”; P=9.39e-06, comparing “chronic” and “pre”).

Significant changes in the genome-wide mCG levels of certain cell types were observed following HIV-1 infection. For instance, memory CD8 T cells exhibited an increase in global mCG levels from “pre” to “chronic” and “acute” stages (Figure S3E). Other cell types such as Memory B cells, Naive B cells, and Naive NK cells also demonstrated significant global mCG changes between the “pre” and “acute” stages (Figure S3E). Subsequently, we identified differentially methylated regions (DMRs) between the “pre,” “acute,” and “chronic” stages for these cell types. Interestingly, highly distinctive methylation patterns were observed between the three stages across all samples (Figure 3B), indicating that these DMRs are highly consistent with disease progression. In total, we identified 104,426 DMRs between the three stages, with 103,460 (99.07%) of them being single CpG sites (Table S2). Single CpG DMRs contained fewer CG contexts within 250 bp compared to other multi-CpG exposure DMRs (Wilcoxon Rank Sum Test, p-value=1.18e-130), while they contained more CGs than both multi-CpG (Wilcoxon Rank Sum Test, p-value=0.014) and single CpG cell type DMRs (Wilcoxon Rank Sum Test, p-value=0.0) (Figure S3F). The largest number of hypomethylated DMRs (hypo-DMRs) was observed in memory CD8 T cells in the “acute” stage, while hypermethylated DMRs (hyper-DMRs) were predominant in the “pre” stage (Figure S3G), consistent with the observed global mCG increase between the “pre” and “acute” and “chronic” stages of infection. Interestingly, the memory state of different cell types, including active NK cells, exhibited more DMRs compared to their naive forms (Figure S3G).

To determine which transcription factors (TFs) may bind to these hypo-DMRs, we identified enriched TF motifs using HOMER (Heinz et al., 2010). ETS family motifs were found to be enriched in most cell types across the three stages of HIV-1 infection, whether in hypo-DMRs or hyper-DMRs (Figure 3C), suggesting a significant rearrangement of ETS family transcription factor binding following HIV-1 infection. For example, ETS family motifs were enriched in the hyper-DMRs of the “acute” and “chronic” stages in monocytes and naive CD8 T cells, while they were also enriched in the hypo-DMRs of the “pre” stage in these cell types (Figure 3C). The enrichment of PU.1 and Fli1 motifs (Figure 3C), which are transcription factors (Minderjahn et al., 2020) involved in regulating immune cell differentiation, suggests that immune cells may be undergoing differentiation into other lineages. RUNX motifs were enriched in hyper-DMRs of the “pre” stage in Naive NK cells, memory and naive CD8 T cells, while also being enriched in hypo-DMRs of the “acute” stage in these cell types. Together with other transcription factors, RUNX1/2 and ETS1 can alter chromatin state (Korinfskaya et al., 2021), potentially influencing T cell lineage determination. RUNX1 and RUNX3 have also been reported to drive the adaptive response of NK cells during MCMV infection (Rapp et al., 2017). Interestingly, motifs of transcription factors associated with circadian function were enriched in hypo-DMRs from the “pre” stage (Figure 3C). These circadian transcription factors include CLOCK, BMAL1, bHLHE41, and NPAS2, suggesting that circadian regulation may be altered in CD8 T cells after HIV-1 infection. Circadian transcription motifs were also enriched in hypo-DMRs of B cells in the “acute” stage. Additionally, we observed CFCF motifs enriched in naive B and naive CD4 T cells, indicating potential 3D genome reorganization in these cell types after HIV-1 infection (Rao et al., 2014; Nora et al., 2017).

Furthermore, we identified differentially methylated genes (DMGs) among the nine cell types between different HIV-1 groups. In pairwise comparisons of the “pre,” “acute,” and “chronic” stages, we identified 288 DMGs (Table S3), with the majority found between the “pre” and “acute” stages in memory CD8 T cells (Figure S3H). Among these DMGs, 112 genes exhibited hypomethylation in the “pre” stage, while 69 genes showed hypomethylation in the “acute” stage. Both groups of DMGs were enriched in genes with immune-related functions (Figure S3I).

To validate the reliability of these methylation signatures and investigate potential regulatory mechanisms, we integrated DNA methylation data with single-cell ATAC-seq data. We mapped the cells from single-cell ATAC-seq to methylation clusters and transferred the cluster labels using Canonical Correlation Analysis (CCA) (Stuart et al., 2019). To validate our integration approach, we calculated the genome-wide correlation between these two modalities (Figure 3D). Specifically, we divided the genome into 5 kb bins and calculated the hypomethylation score using single-cell methylation data and the number of Tn5 insertions using single-cell ATAC data (Liu et al., 2021). We then calculated the correlation between these two measurements for each bin. We observed a strong correlation between the two modalities (methylation and open chromatin) across all cell types, with the highest correlation observed in monocytes (Figure 3D). Methylation in NK cells also showed a high correlation with chromatin accessibility in T cells (Figure 3D). Interestingly, we detected a loss of methylation and an increase in accessibility after HIV-1 infection in memory CD8 T cells at the intron of DGKH (Figure 3E), a gene previously reported to exhibit differential methylation between elite controllers (individuals able to maintain undetectable viral loads for at least 12 months without antiretroviral therapy) and individuals receiving antiretroviral therapy (Frias et al., 2021). Although this region experienced a loss of chromatin accessibility, the methylation level remained unchanged between the “acute” and “chronic” stages (Figure 3E). When comparing all DMRs with differentially accessible regions (DARs) from the two modalities, we found a minimal overlap of only about 2%, highlighting the necessity of using both technologies to profile the epigenomic remodeling associated with HIV-1 infection.

### Impact of influenza infection on the methylome

To investigate how influenza infection can affect the methylome of immune cell types, we conducted experiments examining PBMCs collected from influenza virus-infected volunteers before and 28 days after infection, as part of an experimental influenza vaccine trial (Figure 1). Using single-cell methylation sequencing on these cells, we identified 11 cell clusters (Figure S4A) and further annotated them into 9 cell types based on cell surface markers and methylation profiles (Figure 3F). The distribution of cells from all patients was even among the clusters, indicating the absence of significant batch effects (Figure S4B). Similar to the control and HIV cohorts, we observed two sub-clusters of B cells and NK cells, but the ratio between these subclusters varied significantly compared to the other cohorts. The ratio between B-Memory and B-Naive cells was 0.54:1, while the ratio between NK-Active and NK-Naive was 0.3:1 (Figure S4D). Interestingly, NK-Active cells clustered together with memory CD8 T cells, which also exhibited lower global mCG levels (Figure 3F), suggesting similarity between NK and memory CD8 T cell methylomes.

The global mCG levels of the nine cell types did not show significant differences between the ‘pre’ and ‘post’ groups, except for some moderate changes in NK-Naive cells (Figure S4E). Furthermore, no differentially methylated genes (DMGs) were identified between the “pre” and “post” groups, indicating that there were no drastic changes in gene body methylation 28 days after infection. To further characterize the genomic regions whose methylation is associated with influenza infection, we identified differentially methylated regions (DMRs) between the ‘pre’ and ‘post’ stages of influenza infection in all cell types. We found 71,995 DMRs associated with influenza infection in all cell types (Table S4), with the vast majority (99.49%) being single CpG sites in CD4 memory T cells (Figure S4F). These DMRs were equally distributed between hypo- and hyper-DMRs (Figure S4F) and exhibited distinct patterns between the two groups (pre vs. post) (Figure 3G).

To determine the potential transcription factors that could bind to these DMRs, we identified enriched motifs (Heinz et al., 2010). ETS motifs were significantly enriched in both hypo- and hyper-DMRs from the ‘pre’ and ‘post’ stages in almost every cell type, except for memory B cells, active NK cells, and memory CD8 and CD4 T cells (Figure 3H), suggesting rearrangement of ETS family transcription factor binding sites after influenza infection. Transcription factors such as PU.1, ETS1, and Fli1 were enriched in naive B and T cells, indicating potential differentiation of these naive immune cells after influenza infection. RUNX motifs were enriched in hypo-DMRs after influenza infection in naive NK cells, suggesting activation of these cells (Rapp et al., 2017). However, in naive CD8 and CD4 T cells, RUNX motifs were enriched in both hypo- and hyper-DMRs in the ‘pre’ and ‘post’ stages (Figure 3H). Circadian transcription factor motifs were also enriched in hypo-DMRs from the ‘pre’ stage in memory and naive CD8 T cells, while they were enriched in hypo-DMRs from memory CD4 T cells after influenza infection. We also observed enrichment of MEF2A, MEF2B, and MEF2C motifs in hypo- and hyper-DMRs before influenza infection in memory B and CD8 T cells (Figure 3H). Interestingly, MEF2 is necessary for the transcriptional activation of certain cytokines during peripheral T-cell activation (Pan et al., 2004). Similar to HIV-1 infections, the CTCF motif was enriched in naive B cells, monocytes, and NK-Naive cells, indicating potential 3D genome reorganization of these cell types after influenza infection (Rao et al., 2014; Nora et al., 2017).

To verify the quality of our single-cell methylation data and further validate the identified DMRs, we integrated the methylation data with the single-cell ATAC-seq data and transferred the cell type labels to the single-cell ATAC-seq cells. We calculated the genome-wide correlation between the hypomethylation score and the number of Tn5 insertions using 5 kb bins with the single-cell methylation and ATAC-seq data. The two modalities showed high correlation in B cells, monocytes, and NK cells, while the correlation was lower in T cells (Figure S4G).

### Impact of SARS-CoV-2 Infection

**Figure 4.**
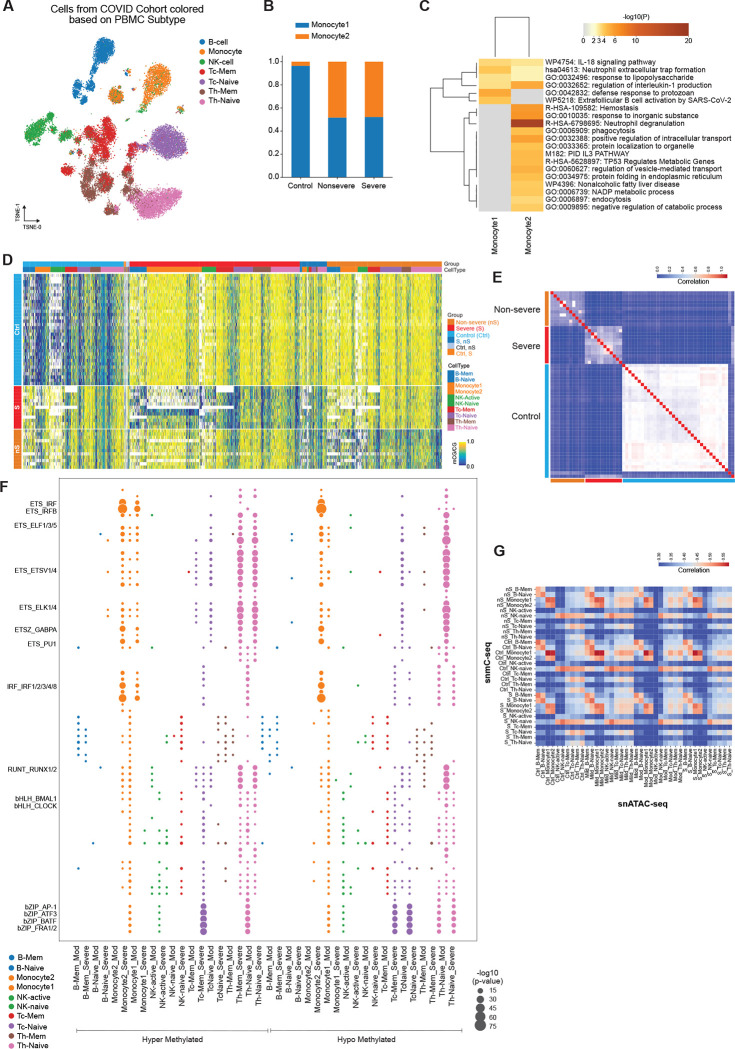
Impact of SARS-CoV-2 infection on the methylomes of immune cells. **A.** t-SNE embeddings of single-nucleus methylomes from the COVID-19 cohort, together with the healthy controls from this study. Colors indicate cell surface markers. **B.** Bar plot showing the proportion of cells from two clusters of monocytes. A chi-square test was used to determine the enrichment of moderate or severe COVID-19 samples in these two clusters of monocytes. **C.** Functional enrichment of differentially methylated genes between the two clusters of monocytes. **D.** Heatmap showing the methylation level at DMRs between moderate, severe, and control samples. The color scale represents methylation levels. DMRs are grouped by hypomethylation and further grouped by cell types. E. Heatmap showing the correlation of samples from moderate, severe, and control groups at DMRs between the three groups. **F.** Scatter plot showing enriched motifs of DMRs between moderate, severe COVID-19 samples and controls in all cell types. The color represents cell types defined by cell surface markers, while the size of the dots represents the enrichment p-value. **G.** Heatmap showing genome-wide correlations between pseudo-bulk signals from each cell type in single-nucleus methylation and matched single-nucleus ATAC-seq. Red indicates stronger correlation, while blue indicates weaker correlation.

The SARS-CoV-2 virus, the pathogen causing COVID-19, continues to be a major global public health concern. However, little is known about the impact of SARS-CoV-2 infection on the dynamics of DNA methylation in immune cells. To examine this, we profiled the methylome of immune cells as previously described. We collected samples from patients with severe and non-severe disease and identified 21 cell clusters (Figure S5A), which were annotated to 10 cell types based on both cell surface markers and methylation profiles. In addition to two sub-clusters of B cells (B-Mem, B-Naive) and NK cells (NK-Active, NK-Naive), we also identified two populations of monocytes (Figure 4A), present in both severe and non-severe disease patients (Figure S5B). Compared to the subclusters of B cells and NK cells, the genome-wide global mCG levels of these two clusters of monocytes are more similar (Figure S5C). The ratios of cells in these two clusters of monocytes are comparable between severe and non-severe samples, while control monocytes are significantly enriched in one of the clusters (Figure 4B) (P=2.05e-237, Chi-square test). We refer to the cluster of monocytes that are rare in control samples as ‘Monocyte2’, and the monocyte cluster that is abundant in control samples as ‘Monocyte1’, a cluster also identified in other exposures. In addition to monocytes, the cell ratios between the two clusters of NK cells and B cells are also significantly different between non-severe, severe, and control samples (Figure S5D, S5E) (P=2.76e-20, Chi-square test, comparison between B-Mem and B-Naive; P=3.51e-05, Chi-square test, comparison between NK-Active and NK-Naive).

To compare the differences between the two clusters of monocytes, we identified the differentially methylated genes (DMGs) and differentially methylated regions (DMRs) between them. We identified 321 DMGs between ‘Monocyte1’ and ‘Monocyte2’, of which 262 and 59 genes are hypomethylated in ‘Monocyte2’ and ‘Monocyte1’, respectively (Figure S5D, Table S5). Methylation levels at these genes show distinctive patterns between the two clusters of monocytes (Figure S5D). We also identified DMRs between the two clusters of monocytes, resulting in 118,186 DMRs, about 64,195 of which were single CpG sites (Table S6). Methylation levels at these DMRs in ‘Monocyte1’ and ‘Monocyte2’ across control, non-severe, and severe COVID-19 patients showed high reproducibility (Figure S5G). We further examined gene functional enrichment of these DMRs for each cluster and found that hypo-DMRs in ‘Monocyte2’ are enriched in ‘myeloid cell activation’ and ‘leukocyte degranulation’, while hypo-DMRs in ‘Monocyte1’ are enriched in functions related to ‘abnormal circulating IL-1 level’ (Figure S5H). IL-1 has a pivotal role in the induction of cytokine storms due to uncontrolled immune responses in SARS-CoV-2 infection (Mardi et al., 2021). IL-1 inhibition improved clinical outcomes of severe COVID-19 patients, and this treatment is one of the CDC COVID-19 treatment guidelines.

To compare the methylomes of these immune cell types, we first examined global mCG between control, non-severe, and severe COVID-19 samples. We found that the global mCG was significantly different in the two clusters of monocytes, naive NK cells, naive CD4, and CD8 T cells (Figure S5I). To further determine the methylation signatures associated with non-severe or severe COVID-19, we identified the DMRs in each of the ten immune cell type clusters. In total, we identified 203,809 DMRs, of which 84.06% (171,319) are single CpG sites (Table S7). Consistent with the significant change of global mCG level in ‘Monocyte1’, this sub-cluster had the largest number of DMRs compared to all other cell types, being hypomethylated in severe COVID-19 patients compared with control samples (Figure S5J), indicating dynamic reshaping of the methylome in severe COVID-19 patients. Comparison of the methylation levels at these DMRs in control non-severe and severe COVID-19 samples revealed distinctive patterns between patient groups (Figure 4D). Further correlation analysis of DMRs between these samples showed a higher correlation between non-severe and control samples than between non-severe and severe COVID-19 patients (Figure 4E). To further verify that the DMRs are associated with COVID-19, we shuffled the labels of the samples and performed DMR analysis between the randomly grouped samples, which showed that the DMRs are less distinctive compared to the DMRs between COVID-19 samples and controls (Figure 4K).

To further identify the transcription factor activities associated with non-severe or severe COVID-19, we examined the enrichment of DNA binding motifs at DMRs in the ten immune cell clusters. We found that control hypo-DMRs in Monocyte1, which are also mostly hypermethylated in severe and non-severe COVID-19 samples, are significantly enriched in IRF and ETS motifs (Figure 4F). The ETS family motifs are also enriched in the control hypo-DMRs and COVID-19 hyper-DMRs in naive CD4 and CD8 T cells (Figure 4F). AP-1 family transcription factors are specifically enriched in the hypo-DMRs in COVID-19 patients and hyper-DMRs in controls in naive CD4 and CD8 T cells (Figure 4F), suggesting the activation of these cells after SARS-CoV-2 infection (Yukawa et al., 2020). These AP-1 motifs are also enriched in hypo-DMRs in monocytes in severe COVID-19 patients, and loss of methylation at AP-1 binding sites was also reported from monocyte-to-macrophage differentiation (Dekkers et al., 2019), indicating the potential differentiation of these monocytes. bHLH transcription factors, which include the circadian genes, are also enriched in most of the DMRs in different cell types, with their motifs being enriched in T cells in HIV-1 and influenza cohorts.

As described above, we performed integration between our methylation data and single-cell ATAC-seq data. In order to validate our integration approach and data quality, we calculated the genome-wide correlation between these two modalities (Figure 4G). We observed that the two modalities (methylation and open chromatin) were strongly correlated across all cell types, with the strongest correlation observed in monocytes and the lowest correlation in different types of T cells (Figure 4G). Surprisingly, only ~5% of the DMRs in each cell type overlapped with a peak in the corresponding cell type in ATAC-seq data, indicating that most of the methylation changes associated with COVID-19 are in inaccessible chromatin.

### Impact of MRSA and MSSA infection on the methylome

**Figure 5.**
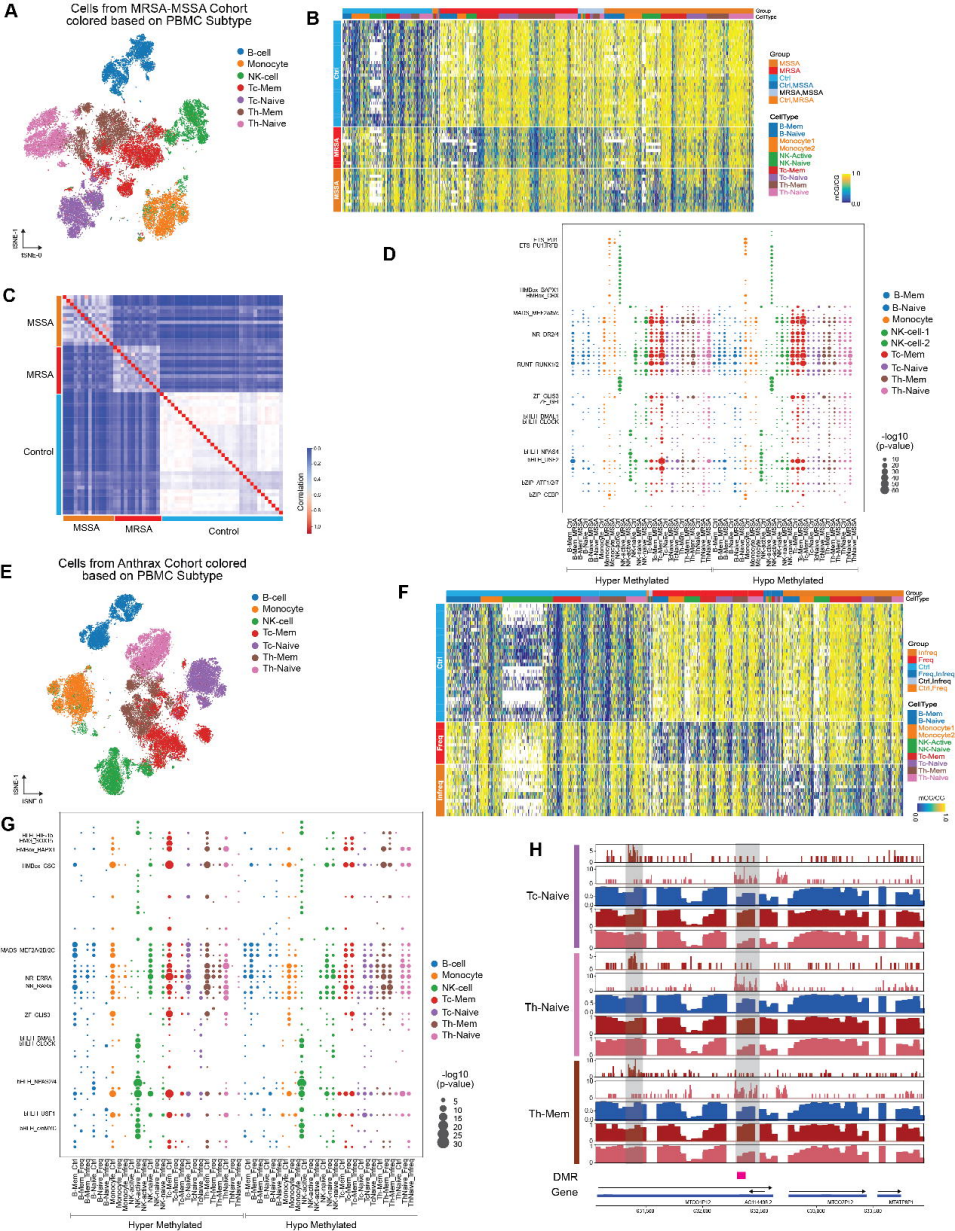
Impact of MRSA/MSSA and BA on the methylome of immune cells. **A.** t-SNE embeddings of single-nucleus methylomes from the MRSA/MSSA cohort, together with the healthy controls from this study. Color indicates the cell surface markers. **B.** Heatmap showing the mCG level at DMRs between MRSA, MSSA, and control samples. The color scale represents methylation levels. DMRs are grouped by hypomethylation and further grouped by cell types. **C.** Heatmap showing the correlation of samples from MRSA, MSSA, and control groups at DMRs between the three groups. **D.** Scatter plot showing enriched motifs of DMRs between MRSA, MSSA, and control samples in all cell types. The color represents cell types defined by cell surface markers, while the size of the dots represents the enrichment p-value. **E.** t-SNE embeddings of single-nucleus methylomes from the BA cohort, together with the healthy controls from this study. Color indicates the cell surface markers of these cells. **F.** Heatmap showing the mCG level at DMRs between frequent, infrequent, and control samples. The color scale represents methylation levels. **G**. Scatter plot showing enriched motifs of DMRs between frequent, infrequent, and control samples in all cell types. The color represents cell types defined by cell surface markers, while the size of the dots represents the enrichment p-value. **H.** Genome browser view of pseudo-bulk single-nucleus ATAC and methylation signals from T cells at gene MTCO1P12. One highlighted region shows an associated gain of accessibility and loss of DNA methylation, while the other region shows a gain of methylation and loss of accessibility.

Methicillin-resistant Staphylococcus aureus (MRSA) and methicillin-susceptible Staphylococcus aureus (MSSA) are both caused by the same strain of bacteria which differ in their susceptibility to antibiotics. To better understand the impact of this infection on the immune cell methylome, we performed snmC-seq2 on the MRSA-MSSA cohort described above. Based on the global methylation profile of the cells, 25 clusters were identified, which were annotated to 9 cell types based on both cell surface markers and methylation profiles (Figure 5A, S6A). These included two sub-clusters of B cells and NK cells, which were labeled as, B-Mem, B-Naive, NK-Active, and NK-Naive, respectively (Figure S6C). These clusters were distributed evenly among MRSA, MSSA patients, and control samples (Figure S6B). The number of cells in the two sub-clusters of NK cells was comparable in the three cohorts (Figure S6D), while we captured more memory B cells in the MSSA cohort compared to the other two (Figure S6D) (P=9.36e-10, Chi-square test).

We observed large changes in global mCG levels between MRSA, MSSA, and controls in all nine cell types (Figure S6E). To further characterize the methylation signatures correlated with MRSA or MSSA infection, we identified DMRs between MRSA, MSSA, and control samples. In total, we identified 134,868 DMRs between the three groups, the majority of which were from memory CD8 T cells (Figure S6F, Table S8), which also showed a significant change in global mCG (Figure S6E). Similar to other exposures, the majority (93.46%) of DMRs were single CpG sites. Visualization of the methylation levels at these DMRs demonstrated high reproducibility, showing a clear distinction between patient and control samples (Figure 5B). The correlation of the methylation level of samples from the three cohorts at these DMRs confirmed that the samples from the three groups are very distinctive from each other at the DMRs (Figure 5C). To further validate that the MRSA or MSSA-associated DMRs we identified are not caused by heterogeneity between individuals, we shuffled the labels of the samples as described above and identified the DMRs between these random groups. The result showed that the DMRs between random groups are much less distinctive than MRSA or MSSA-associated DMRs (Figure S6G).

To identify transcription factors that can potentially bind at these DMRs, we investigated the enrichment of DNA binding motifs. Unlike the virus infections, both RUNX and ETS motifs are only enriched in the hypo-DMRs of MSSA and hyper-DMRs in controls in active NK cells, with some of these motifs enriched in hypo-DMRs of controls and hyper-DMRs of MRSA in monocytes (Figure 5D). Homeobox and MADS family motifs are generally in all cell types and in both hypo- and hyper-DMRs, indicating reshuffling of the binding sites after MRSA or MSSA infections.

### Impact of frequency of exposures in high-risk facilities on the methylome

Bacillus anthracis (BA) is the causative agent of a serious infectious disease called anthrax. In our BA cohort, we collected peripheral blood mononuclear cells (PBMCs) from individuals who received BA vaccines and work either frequently or infrequently in Biosafety Level 3 (BSL3) facilities, handling infectious pathogens or chemicals. Methylation profiles of immune cells identified 23 clusters, further categorized into 9 cell types based on cell surface markers and methylation profiles (Figure 5E, S7A). Immune cells from technicians who handled anthrax, both frequently and infrequently, clustered together across all cell types (Figure S7B). Based on global mCG levels (Figure S7C), we identified two clusters of B cells (B-Mem and B-Naive) and two clusters of NK cells (NK-Active and NK-Naive). The ratio of NK-Active cells in the ‘frequent’ or ‘infrequent’ groups was significantly lower than in the control samples (P=1.74e-64, Chi-square test), while the ratio of memory and naive B cells was comparable across the three cohorts (Figure S7D).

Significant differences in global mCG levels were observed among these immune cell clusters across the three groups (Figure S7E). To explore the epigenetic characteristics of immune cells in individuals handling anthrax frequently or infrequently in BSL3 facilities, we identified differentially methylated regions (DMRs) between these groups compared to a control population. In total, we found 76,757 DMRs among the three groups, with 72,837 (95.12%) consisting of single CpG sites (Table S9). Control samples displayed the highest number of hypo- or hyper-DMRs in all cell types (Figure S7F), suggesting that the observed differences were mainly between the control and BA cohorts. Visualization of the methylation levels at these DMRs clearly distinguished the two groups and the control cohort (Figure 5B). We conducted a permutation test similar to the one described earlier and found that DMRs between random groups were less distinctive (Figure S7G). The correlation of methylation levels at these DMRs in donors from the three groups also exhibited higher correlations within groups (Figure S7H).

To identify potential transcription factors that could bind to these DMRs, we performed an enrichment analysis of DNA binding motifs for each group. Unlike viral infections and similar to the MRSA and MSSA cohort, both RUNX and ETS motifs were not enriched in the DMRs across all cell types (Figure 5G). Circadian transcription factors showed significant enrichment only in hypo-DMRs from control samples in active NK cells (Figure 5G). Homeobox family transcription factor motifs were enriched in hypo-DMRs in active NK cells from control samples (Figure 5G). These homeobox genes act as transcriptional regulators of embryonic development and cell differentiation and have also been reported to regulate NK cell function (Sunwoo et al., 2008; Wu, Zhang, and Zhang, 2011), suggesting potential immune cell differentiation.

As mentioned earlier, we integrated our methylation data with single-cell ATAC-seq data. To validate our integration approach and data quality, we calculated the genome-wide correlation between these two modalities. Similar correlations to other exposures were observed between the two modalities across all cell types (Figure S7I). Interestingly, we observed simultaneous loss of methylation and gain of accessibility, as well as gain of methylation and loss of accessibility, in the ‘frequent’ group at a locus containing a long non-coding RNA (lncRNA) and a pseudogene (Figure 5H). Only approximately 5.5% of the DMRs in each cell type overlapped with a peak in the corresponding cell type in the ATAC-seq data, indicating that most of the methylation changes associated with working frequency in high-risk facilities occur in closed chromatin regions.

### Impact of organophosphate exposure on the methylome

**Figure 6.**
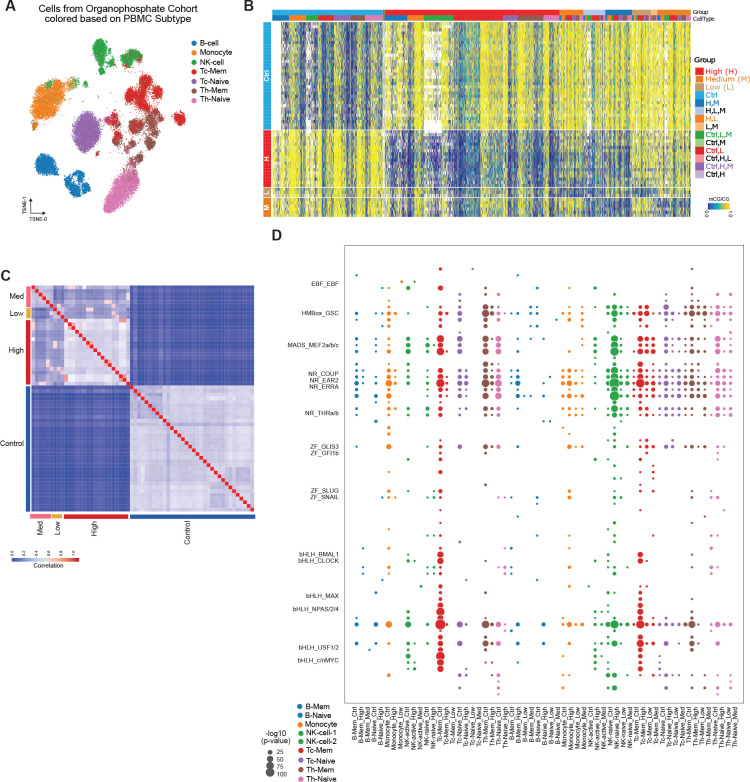
Impact of OP infection on the methylome of immune cells. **A.** t-SNE embeddings of single-nucleus methylomes from the OP cohort, together with the healthy controls from this study. Color indicates the cell surface markers of these cells. **B.** Heatmap showing the mCG level at DMRs between low, median, high OP exposures, and control samples. The color scale represents methylation levels. DMRs are grouped by hypomethylation and further grouped by cell types. **C.** Heatmap showing the correlation of samples from low, median, high OP exposures, and control groups at DMRs between the four groups. **D.** Scatter plot showing enriched motifs of DMRs between low, median, high OP exposure, and control samples in all cell types. The color represents cell types defined by cell surface markers, while the size of the dots represents the enrichment p-value.

Organophosphates (OP) are common insecticides that can harm humans and animals if exposed for too long or at high levels. However, little is known about how OP can affect the methylome of immune cells when exposed to different levels. We performed snmC-seq2 on the peripheral blood mononuclear cells (PBMCs) of individuals exposed to OP at varying levels (Low, Med, and High). These immune cells clustered into 22 clusters and were further annotated into 9 cell types based on both cell surface markers and methylation profiles (Figure 6A, S8A). Cells from patients with different levels of OP exposure were evenly distributed across the cell types (Figure S8B). Similar to other exposures, we identified B-Mem, B-Naive, NK-Active, and NK-Naive based on their global methylation levels (Figure S8C). The proportions of the two clusters of B cells were comparable across different levels of exposure, while the exposure groups had significantly more NK-Active cells compared to control samples (Figure S8D) (P=1.44e-56, Chi-square test).

We explored the association of methylation changes with different levels of OP exposure and observed significant changes in the global mCG levels in most of the cell types (Figure S8E). We further identified differentially methylated regions (DMRs) between the different exposure groups and control samples in all nine cell types, resulting in 198,807 DMRs among the four groups (Low, Med, High, and Control) (Table S10). Memory CD8 T cells had the highest number of DMRs compared to other cell types (Figure S8F). Visualization of the methylation levels at these DMRs showed distinct methylation patterns between these groups (Figure 6B), particularly between OP exposure and control. We performed a similar permutation test as described above and found that the DMRs between random groups were much less distinctive (Figure S8G). The correlation analysis of these samples at the DMRs revealed weak distinctions between the ‘Low’, ‘Med’, and ‘High’ group samples, while the distinction between control samples was evident (Figure 6C).

To further investigate which transcription factors might be affected in these nine cell types, we determined the enriched motifs at these DMRs across all cell types. The results showed that hyper-DMRs in control samples and hypo-DMRs in OP exposure groups were enriched in MADS, RN, and Homeobox family transcription factor motifs in all cell types (Figure 6D), indicating that the impact of this chemical exposure on the methylome does not exhibit cell type specificity.

Similarly, we performed integration between our methylation data and single-cell ATAC-seq data. To validate our integration approach and data quality, we calculated the genome-wide correlation between these two modalities. We observed similar correlations to other exposures between the two modalities in all cell types (Figure S8H). Consistent with other exposures, the majority of DMRs associated with OP were located in inaccessible chromatin regions.

### Comparison of methylation signatures from all exposures

**Figure 7.**
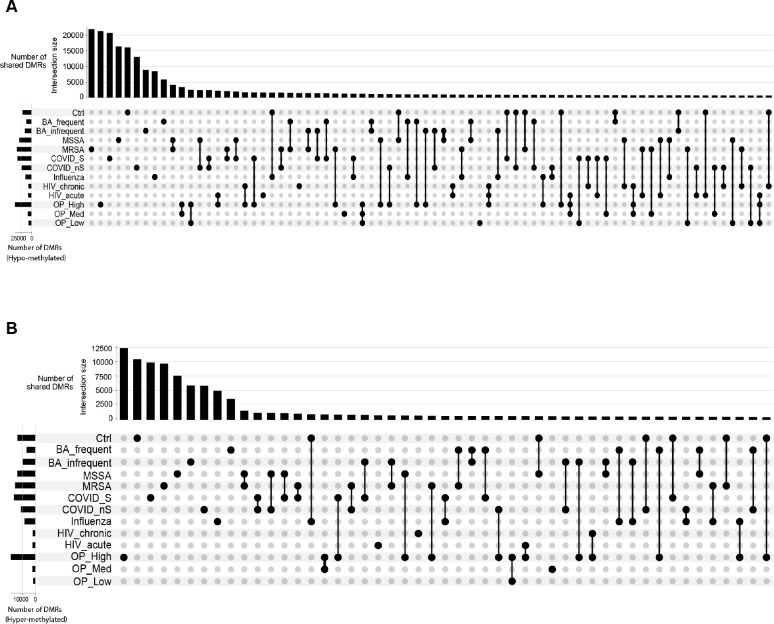
Integrative analysis of DMRs from all exposures. **A.** Upset plot showing the hypo-DMRs shared between different exposures. **B**. Upset plot showing the shared hyper-DMRs between different exposures.

To compare the methylation signatures across different exposures, we merged the methylation files from the same exposure and identified the differentially methylated regions (DMRs) among all the groups using methylpy. This strategy does not rely on control samples and allows for a general comparison of the methylation levels across all the samples. For instance, in Naive B cells, we identified a total of 188,563 hypo-DMRs across all exposures, most of which were unique to a specific exposure (Figure 7A). Interestingly, MRSA and MSSA shared more DMRs than any other two exposures, which aligns with the similarities observed between these two exposures (Figure 7A). We also observed that different levels of OP exposures shared more hypo-DMRs compared to other comparisons, indicating a similar effect of varying levels of OP exposures. However, bacterial exposures and virus exposures did not show a higher number of shared DMRs within the group compared to between groups, as MRSA/MSSA and COVID-19 samples shared more DMRs than any two exposures within bacteria or virus (Figure 7A). We performed a similar analysis for hyper-DMRs among all exposures, which yielded comparable results to the hypo-DMRs (Figure 7B). We applied the same analysis to all other cell types, which demonstrated similar patterns of similarities in DMRs among all exposures for both hypo-DMRs (Figure S9) and hyper-DMRs (Figure S10).

### Predicting exposure types with single-cell methylomes

**Figure 8.**
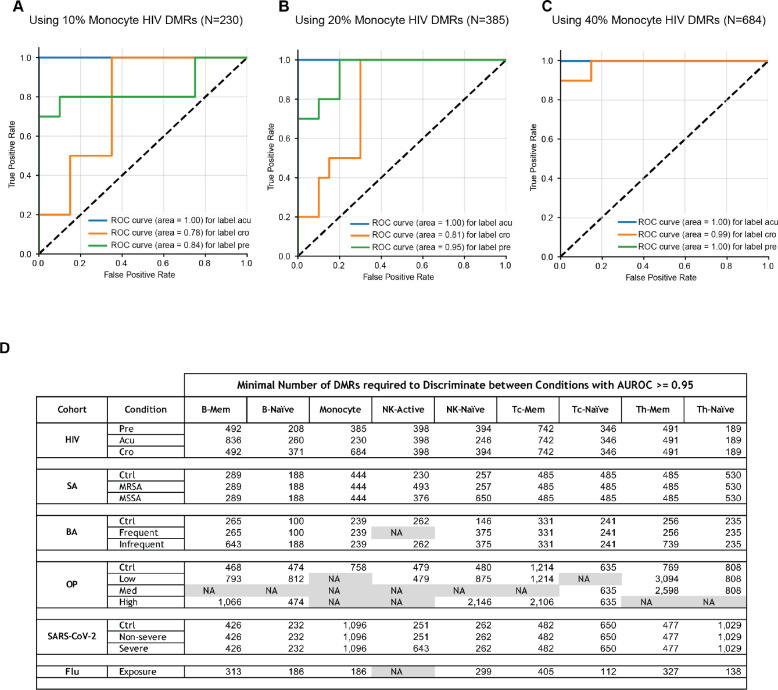
Prediction of different exposures based on methylation signatures. **A**. ROC curve of HIV-1 stages prediction using 714 DMRs (10%) in Monocytes. **B**. ROC curve of HIV-1 stages prediction using 1428 DMRs (20%) in Monocytes. **C**. ROC curve of HIV-1 stages prediction using 2856 DMRs (40%) in Monocytes. **D.** The minimum number of DMRs required in each cell type to discriminate different exposures with AUC > 0.95.

Considering that 790,662 differentially methylated regions (DMRs) were associated with various exposures in the nine immune cell types, we hypothesized that some of these DMRs could accurately predict different exposures. We conducted multiple rounds of training to identify the optimal number of DMRs for accurate prediction. As an example, we used DMRs associated with HIV-1 infection to predict its three stages (pre, acute, chronic). We divided the individual donors into training and test cohorts and created 150 and 50 pseudo-individuals by combining single cells through permutation. Using logistic regression, we trained a model with the 150 pseudo-individuals in the training cohort for each cell type and tested the model with pseudo-individuals in the test cohort. Remarkably, the model was able to perfectly predict the different stages of HIV-1 infection. Next, we aimed to minimize the number of DMRs required for accurate prediction by selecting the top weighted DMRs in the primary prediction at the 10% quantiles. Surprisingly, we achieved perfect prediction of different HIV-1 infection stages using only 30% of the DMRs, and the pre-stage could be predicted with just 10% of the DMRs (Figure 8A–C).

We then expanded the prediction analysis to other exposures, and the results demonstrated that we could predict most exposures with an area under the curve (AUC) greater than 0.95 in receiver operating characteristic (ROC) curves using only a few thousand DMRs (Figure 8D). The minimum discriminative DMRs are provided in Table S11, which can serve as biomarkers for diagnosing different exposures using readily accessible cells.

## Discussion

Here, we conducted an analysis of single-cell methylomes in major immune cell types from PBMCs obtained from both healthy donors and patients exposed to bacteria, viruses, and chemicals. In total, we analyzed the methylomes of 104,876 high-quality cells representing seven major immune cell types. This dataset serves as a valuable resource for studying the epigenomes and epigenetic features of immune cells in response to various exposures. Notably, this study represents the first human immune cell methylome atlas at single-cell resolution. Using this comprehensive resource, we identified differentially methylated regions (DMRs) associated with each exposure and developed unsupervised models to predict exposure based on these DMRs. These findings enhance our understanding of the pathogenesis of various exposures and establish DMRs as potential signatures of different exposures, which can aid in the diagnosis using methylation markers.

The diagnosis of different exposures based on selected CpG sites in immune cells has broad applications in public health and can be accomplished using our prediction model. In addition to providing biological insights into different exposures, the DMRs that exhibit high predictive weights for different exposures can serve as biomarkers for infectious disease diagnosis, offering a valuable clinical resource. Host methylation signatures have already been utilized for predicting SARS-CoV-2 infections (Konigsberg et al., 2021), indicating that methylation markers can be independently identified in other cohorts. Recent investigations have extensively explored host DNA methylation in virus and bacterial infections, revealing associations between methylation markers and various infections (Qin, Scicluna, and van der Poll, 2021). For instance, a study discovered methylation signatures associated with persistent MRSA (Chang et al., 2021), while another study demonstrated that a bacterial infection can remodel the methylation profile of dendritic cells (Pacis et al., n.d.). In our study, we identified signatures at single-cell resolution in a cell type-specific manner, extending the diagnostic potential to multiple exposures, including viruses, bacteria, and chemicals.

The majority of DMRs associated with exposures (~99% for most exposures) comprise single CpG sites primarily located in intergenic and inaccessible regions. This is in contrast to the 36% of single CpG DMRs observed between different cell types in control cohorts. Genomic analysis revealed that exposure-associated single CpG DMRs generally have fewer CGs compared to multi-CpG exposure DMRs. However, in some exposures, single CpG DMRs even exhibit a higher number of CGs than multi-CpG cell type DMRs (Figure S3F, Figure S11). This suggests that the density of CGs alone cannot explain the substantial proportion of single CpG DMRs observed in each exposure. Consequently, it implies the involvement of novel methylation or demethylation mechanisms in the infection-induced changes in single CpG DNA methylation. Single CpG methylation or demethylation is known to occur and has regulatory functions. Previous studies have demonstrated that methylation of single CpG sites can regulate gene expression (Zhang et al., 2010; Wang et al., 2011), while demethylation of single CpG sites can trigger gene expression and regulate splicing (Ceccarelli et al., 2011; Venza et al., 2012; Sobiak and Leśniak, 2019).

One of the major signatures of the immune cells in these exposures is that the cell proportions of the two B cell and NK cell clusters vary in different exposures. These B cells and NK cells are sorted from PBMCs, and there can be some bias in the sorting process. However, the proportions of the two subtypes in B cells and NK cells we identified with single-cell methylation profiles can reflect their relative abundance in PBMCs. The relative abundance of naive and memory B cells varies at different ages (Morbach et al. 2010), suggesting that the different age compositions of the donors from different exposures might contribute to the variabilities of B cell subtypes. However, there are significant changes in B cell subtypes during infections like SARS-CoV-2 (Sosa-Hernández et al. 2020). Alterations of NK cell subtype frequencies were also reported in HIV and HCV infections (Meier et al. 2005), indicating that the relative abundance of the B cell and NK cell subtypes might be associated with different exposures.

A unique cluster of monocytes specific to COVID-19 samples confirms the association of these monocytes with COVID-19. Single-cell RNA-seq studies on COVID-19 samples have also found that classical monocytes are the main source of major COVID-19 mediating cytokines (Vanderbeke et al., 2021; N. Liu et al., 2021). Our studies identified the cytokines mediated by monocytes based on single-cell epigenomes. The epigenetic features associated with this unique cluster of monocytes can provide insights into the mechanisms of the cytokine storm related to monocytes. Considerable changes in methylation were also observed in the common monocyte cluster in COVID-19 patients, indicating that the two clusters of monocytes are unique in SARS-CoV-2 infection.

Specific TF binding motifs were found to be enriched in the differentially methylated regions (DMRs) associated with each exposure, suggesting exposure-specific changes in transcription factor activities across distinct cell types. TFs known to regulate cell differentiation and function, such as RUNX, ETS family transcription factors, and PU.1, were also enriched at certain DMRs.

These TFs play key roles in the function and differentiation of immune cells (Sharrocks 2001; Korinfskaya et al. 2021; Hollenhorst, McIntosh, and Graves 2011; Hosokawa and Rothenberg 2020). ETS and RUNX motifs were generally enriched across most cell types in the virus exposures, but only enriched in monocytes and active NK cells in the MRSA/MSSA cohort (Figure S12). Circadian transcription factor motifs were enriched in all cohorts, with higher enrichment in T cells for HIV-1, influenza, and OP cohorts, and in NK cells and monocytes for other exposures (Figure S12). This suggests that different exposures may alter the activities of circadian transcription factors, which in turn can impact the functions of immune cells (Hergenhan et al. 2020; Wang et al. 2022). Further studies are needed to explore the specific transcription factors functioning in each immune cell type and investigate their target genes. These studies will contribute to a better understanding of the mechanisms underlying immune reactions to different exposures.

While our study provides valuable resources and insights into infectious diseases and chemical exposure, there are certain limitations to consider. In order to identify methylation features of major immune cell types in an unbiased manner, we focused on sorting seven immune cell types from PBMCs. However, this approach limited our investigation to only these cell types, and we were unable to explore other cell types of interest. Additionally, we analyzed the methylomes of over 100,000 immune cells processed in different batches, which introduced potential batch effects. Although we placed different cell types on the same plate to control for this, some batch-related variability may still exist. Despite applying stringent filtering to identify exposure-associated DMRs, it is possible that we captured individual differences in methylation rather than exposure-related differences. Furthermore, the cohorts were enrolled at different times, and PBMCs were collected and processed by different groups, introducing technical pre-analytic variability specific to each cohort.

### Lead contact

Further information and requests for resources and reagents should be directed to and will be fulfilled by the Lead Contact, Joseph R. Ecker (ecker@salk.edu).

### Materials availability

This study did not generate new unique reagents.

## RESOURCE AVAILABILITY

### Materials availability

This study did not generate new unique reagents.

#### Data and code availability

De-identified molecular data and sample metadata is available through controlled access via dbGaP PHS Accession phs003204.v1.p1.

Codes of all the analysis are available on github (https://github.com/wangwl/ECHO)

## EXPERIMENTAL MODEL AND SUBJECT DETAILS

### Overview of the Cohorts

#### HIV Cohort:

The iPrEx cohort (Iniciativa Profilaxis Pre-Exposición, or the PreP pre-exposure prophylaxis initiative) was a phase III clinical trial aimed at determining the efficacy of pre-exposure prophylaxis (PrEP), an antiretroviral treatment, for preventing HIV infection (Grant et al., 2010). The study enrolled high-risk individuals who were continuously monitored for the onset of HIV-1 infection. During the study, some participants became HIV-1 positive. We obtained samples from nine donors taken approximately ~250 days (median of 258 days) before testing positive for HIV-1 (pre), on the day of the HIV-1 positive blood draw (acute), and approximately ~200 days after treatment (chronic). We observed significant variation in the initial virus load and the load after 200 days during the chronic stage when standard HIV-1 treatment was provided. For this study, we utilized peripheral blood mononuclear cell (PBMC) samples from nine subjects at three time points (see Supplementary Table S1).

#### Influenza Cohort:

The BARDA-Vaccitech FLU010 Study with A/BELGIUM/4217/2015 (H3N2) challenge aimed to assess the protective capabilities of a vaccine candidate, VTP-100, as a standalone influenza vaccine. We obtained pre- and post-challenge samples from 18 donors who received the placebo vaccine (see Supplementary Table S1), with post-challenge sampling conducted 28 days after the challenge dose.

#### COVID-19 Cohort:

Patients who were hospitalized following SARS-CoV-2 infection, with similar COVID severities based on the WHO ordinal severity score between 4 and 7, were enrolled in this cohort. Some patients showed improvement with moderate disease (non-severe group), while others experienced clinical deterioration within a 14-day period (increased severity score) and required invasive mechanical ventilation or succumbed to the disease (severe group). We obtained samples from nine patients in each group (see Supplementary Table S1).

#### MRSA-MSSA Cohort:

We collected samples from 19 patients who tested positive for either Methicillin-resistant Staphylococcus aureus (MRSA) or Methicillin-sensitive Staphylococcus aureus (MSSA). Ten donors were infected with MRSA, while nine donors were infected with MSSA. Among them, nine donors provided two or three longitudinal samples, resulting in a total of 27 samples, with 13 MRSA samples and 14 MSSA samples (see Supplementary Table S1). The time intervals between the second and third time points ranged from 3 to 35 days since the first positive test.

#### Bacillus anthracis Cohort:

We obtained PBMC samples from 27 individuals who were vaccinated against anthrax and worked with Bacillus anthracis in a controlled facility while wearing personal protective equipment (see Supplementary Table S1). These individuals were trained technicians working in Biosafety Level 3 (BSL3) facilities and received vaccination against B. anthracis infection. The vaccine used was Anthrax Vaccine Adsorbed, distributed under the trade name BioThrax^®^. It is an inactivated, acellular vaccine primarily containing the non-pathogenic protective antigen (PA) protein. The vaccine is produced from cell-free filtrates of microaerophilic cultures of an avirulent, non-encapsulated strain of Bacillus anthracis. The donors also handled chemical weapon agents, precursor molecules to chemical agents, biological warfare agents, explosives, precursors to explosives, agricultural chemicals, or radioactive materials in a controlled environment.

#### Organophosphates Cohort:

Organophosphates (OP) are a class of pesticides known to have a severe impact on the dopaminergic and serotonergic systems. One commonly used form of this pesticide, Chlorpyrifos, has extensive use in the United States. Exposure to chlorpyrifos, the most widely used OP in the US, was estimated based on levels of its urinary metabolite 3,5,6-trichloro-2-pyridinol (TCPY) and classified as high, moderate, or low (with undetectable levels of TCPy). In this study, we analyzed DNA methylation patterns in 18 high-exposure, six moderate-exposure, and three low-exposure samples (see Supplementary Table S1).

#### Control Cohorts:

We obtained PBMC samples from 12 healthy donors through a commercial vendor (Dx Biosamples LLC). These donors represented a range of ethnic diversity, age groups, and sexes (see Supplementary Table S1). Together with the pre-HIV samples and the pre-influenza challenge samples mentioned above, we obtained a total of 34 control PBMC samples.

De-identified human PBMC samples were provided by the collaborating teams (listed in the author contributions section). Informed consent was obtained from the donors by the collaborating teams and their respective institutional IRBs.

## Method Details

### Fluorescence-activated Cell Sorting of immune cell types

Cells were sorted into 384-well plates using fluorescence-activated cell sorting (FACS) based on their specific antibody labeling. The FACS antibody cocktail allowed for the identification of seven different immune cell types in blood. The sorted cell types included Naive helper T cells (CD3+, CD4+, CCR7+, CD45RA+), Memory helper T cells (CD3+, CD4+, CD45RA−), Naive cytotoxic T cells (CD3+, CD8+, CCR7+, CD45RA+), Memory cytotoxic T cells (CD3+, CD8+, CD45RA−), B cells (CD3−, CD19+), Monocytes (CD3−, CD19−, CD14+), NK cells (CD3−, CD19−, CD14−, CD16+, CD56+), and other cells (CD3−, CD19−, CD14−, CD16−, CD56−). The SONY Muti-Application Cell Sorter LE-MA900 Series was used to isolate single cells in 384-well PCR plates containing protein kinase. After cell sorting, the plates were spun down to capture the cells at the bottom of the well and then subjected to thermocycling at 50 °C for 20 minutes. The plates containing the DNA from the cells were subsequently stored at −20 °C or moved directly to library preparation.

### Library preparation and Illumina sequencing

For library preparation, we followed the previously described methods for bisulfite conversion and library preparation in snmC-seq2 (Luo et al., 2017, 2018). The snmC-seq2 libraries generated from the isolated immune cells were sequenced using an Illumina Novaseq 6000 instrument with S4 flow cells in the 150-bp paired-end mode. Freedom EVOware v2.7 was utilized for library preparation, while Illumina MiSeq control software v3.1.0.13 and NovaSeq 6000 control software v1.6.0/Real-Time Analysis (RTA) v3.4.4 were employed for sequencing.

## QUANTIFICATION AND STATISTICAL ANALYSIS

### Single-cell methylation data processing (alignment, QC)

For alignment and quality control (QC) of the single-cell methylation data, we employed the same mapping strategy used in our previous single-cell methylation projects in our lab (Liu et al., 2021). Specifically, we utilized our in-house mapping pipeline, YAP (https://hq-1.gitbook.io/mc/), for all the mapping-related analysis. The pipeline includes the following main steps: (1) demultiplexing FASTQ files into single cells, (2) reads-level QC, (3) mapping, (4) BAM file processing and QC, and (5) generation of the final molecular profile. Detailed descriptions of these steps for snmC-seq2 can be found in the work by Luo et al. (2018). All the reads were mapped to the human hg38 genome, and we calculated the methylcytosine counts and total cytosine counts for two sets of genomic regions in each cell after mapping.

We filtered out low-quality cells based on three metrics generated during mapping: mapping rate > 50%, final mC reads > 500,000, and global mCG > 0.5. Chromosomes X, Y, and M were excluded from the analysis, and the remaining genome was divided into 5 kb bins to create a cell-by-bin matrix. In this matrix, each bin was assigned a hypomethylation score (hypo-score) calculated from the p-values of a binomial test, which indicates the probability of hypomethylation of that bin. The matrix was further binarized for downstream analysis using a hypo-score cutoff of >= 0.95. Bins covered by fewer than 5 cells and those with an absolute z-score greater than 2 were filtered out. Additionally, we excluded bins that overlapped with the ENCODE blacklist using “bedtools intersect” (Dale, Pedersen, and Quinlan 2011; Quinlan and Hall 2010).

### Unsupervised clustering

To perform unsupervised clustering, we utilized ALLCools (Liu et al., 2021), which first conducted principal component analysis (PCA) on the 5 kb bin matrix. For each exposure, we selected the top 32 principal components (PCs) for clustering using the modules in scanpy (Wolf, Angerer, and Theis 2018). In the HIV-1 and influenza cohorts, we observed a donor effect in the clustering results with these PCs. Therefore, we applied harmony (Korsunsky et al., n.d.) to correct the donor effect on these PCs. We performed clustering separately for control samples (‘HIV_pre’, ‘Flu_pre’, and ‘Ctrl’) and samples from the ‘MRSA/MSSA’, ‘BA’, ‘COVID-19’, and ‘OP’ groups, allowing for better comparison between the exposures and control samples.

To annotate the cells, we used both the single-cell methylation clustering results and cell surface markers. In almost every cohort, we observed two clusters of B cells and NK cells, which were distinguished by their global mCG levels. Therefore, we assigned these clusters as naive and memory B cells, naive and active NK cells. We also merged clusters with cell surface markers indicating memory CD4 and CD8 T cells, even if they exhibited multiple clusters in the T-SNE embedding.

### Differentially methylated regions (DMR) identification

To identify DMRs associated with each immune cell type, we utilized peripheral blood mononuclear cells (PBMCs) from healthy donors. Based on single-cell methylation and fluorescence-activated cell sorting (FACS), we identified nine cell types through clustering. These cell types were grouped based on their global mCG levels, and DMRs were called separately within high-mCG and low-mCG cell types. We employed methylpy (https://github.com/yupenghe/methylpy) for DMR calling, and the resulting DMRs were further annotated with genes and promoters.

For the identification of DMRs associated with each exposure, we merged the control samples and samples from each exposure group. We used methylpy (https://github.com/yupenghe/methylpy) to identify DMRs between the control and exposure groups, as well as between different exposure groups. Once we obtained the primary set of DMRs, we calculated the methylation levels of all samples at these DMRs using “methylpy add-methylation-level”.

Additional filtering on the DMRs was performed by comparing the methylation levels among different sample groups using Student’s t-test. Only DMRs with a minimum p-value less than 0.05 between any two groups were retained. For DMRs associated with MRSA/MSSA, BA, OP, and SARS-CoV-2, where external controls were used for DMR calling, we compared the methylation levels of exposure samples and control samples, as well as different cohorts of controls (HIV, Flu, and commercial controls). DMRs that showed significant differences (p-value < 0.05) between the exposure group and all three control cohorts, but not significant differences (p-value > 0.05) between any two control cohorts, were retained.

To visualize the complex heatmaps, we employed PyComplexHeatmap (https://github.com/DingWB/PyComplexHeatmap). Hypomethylated DMRs in the corresponding sample groups and cell types were labeled for better visualization. The heatmap rows were split according to sample groups, and the columns were split based on DMR groups and cell types. Within each subgroup, rows and columns were clustered using ward linkage and the Jaccard metric.

### Differentially methylated loci (DML) identification

We used DSS (Park and Wu 2016) to call DMLs for each exposure. The samples were grouped based on exposure types, and pairwise comparisons were made to identify DMLs. To optimize memory and time usage, DSS was run on each chromosome, and the results were merged to calculate the false discovery rate (FDR) using the statsmodels package. We compared the DMRs from methylpy with the DMLs from DSS by examining the heatmaps of mCG levels at these sites in all samples. After evaluation, we decided to use the DMRs from methylpy for downstream analysis.

### Validation of DMRs by shuffling the samples

To validate that the identified DMRs for each exposure were not confounded by batch effects or other factors, we shuffled the group labels of the samples within each exposure and identified DMRs among the randomly assigned groups. We quantified the methylation levels of all samples at the DMRs from the random groups and performed t-tests on the methylation levels between each pair of groups.

### Motif enrichment

We obtained the hypo- and hyper-DMRs reported by methylpy from the columns ‘hypermethylated_samples’ and ‘hypomethylated_samples’. HOMER was used to identify enriched motifs within these different sets of DMRs for each exposure. The results from HOMER’s ‘knownResults.txt’ output files were used for downstream analysis. Only motif enrichments with a p-value < 0.01 were retained. The motif enrichment results were visualized using scatterplots in seaborn.

### Differentially methylated gene (DMG) identification

Pairwise differential methylation analysis of genes (DMGs) for each exposure was performed using ALLCools, following the tutorial (https://lhqing.github.io/ALLCools/cell_level/dmg/04-PairwiseDMG.html). Significantly differentially methylated genes were selected based on an FDR < 0.01 and a delta mCG > 0.05. The functional enrichment analysis of the DMGs was conducted using metascape (Zhou et al., 2019) (https://metascape.org/).

### Functional enrichment of DMRs

To perform functional enrichment analysis of DMLs, we utilized GREAT (http://great.stanford.edu/public/html/index.php).

### Integration with single-cell ATAC

We integrated our single-cell methylation data with single-cell ATAC-seq data from HIV-1, COVID-19, BA, and OP cohorts, as well as single-cell multiome data from influenza. This integration was performed using Canonical Correlation Analysis (CCA), where we transferred our methylation cell annotations to the cells from the other modality. To generate the peaks and bigwig files for each cell type, we utilized SnapATAC2 (Zhang et al., 2021; Fang et al., 2021).

### Correlation of single-cell methylation and single-cell ATAC

To assess the correlation between single-cell methylation and single-cell ATAC, we calculated the correlation between the hypo-score of each 5 kb bin and Tn5 insertions in each bin. This correlation was performed between cell types and within matched cell types.

### Prediction of exposures using sub-clusters, genes, and DMRs

To predict different exposures, we utilized three pieces of information: sub-clusters, genes, and differentially methylated loci (DMLs).For sub-cluster prediction, we performed clustering within each sorted cell type from all exposures and identified sub-clusters based on Leiden clustering. We divided the samples into training and test sets at a ratio of 6:4. Then, we generated 1000 pseudo-individuals by permuting the training and testing samples (700 as training and 300 as the test set). From each pseudo-individual, we randomly sampled 500 cells and calculated their distribution across the sub-clusters. We used this distribution to predict different exposures.

For gene-based prediction, we utilized the methylation level of genes. For each cell type, we generated 400 pseudo-individuals, with 300 as training samples and 100 as the test set. The samples were divided into training and testing sets at a ratio of 7:3. We calculated the gene body methylation level for all genes and used this information to predict different exposures.Similarly, for DML-based prediction, we utilized DMLs to predict different exposures. The process was similar to the gene-based prediction, where we quantified the DMLs by methylation level and used this information to predict different exposures within each cell type.

## Supplementary Material

Supplement 1

Supplement 2

Supplement 3

Supplement 4

Supplement 5

Supplement 6

Supplement 7

Supplement 8

Supplement 9

Supplement 10

Supplement 11

Supplement 12 **Figure S1. FACS gating strategy for selecting seven major immune effector cell types.** SSC: Side Scatter; FSC: Forward Scatter. Blue arrows indicate further classification of the population. The red arrows and red font indicate the sampled cell. The cell surface antibody marker for each subtype is underlined in the axes, and the values on the axes indicate the fluorescence wavelength used.

Supplement 13 **Figure S2. Quality control of single-cell analysis. A**. Distribution of genome coverage with reads from each cell. The X-axis shows the coverage of the genome, and the Y-axis shows the number of cells with that coverage. **B**. Distribution of the mapping rate of reads from each cell. The X-axis shows the mapping rate of the reads, and the Y-axis shows the number of cells with that mapping rate. **C.** Distribution of the final mC reads from each cell. The X-axis shows the number of final mC reads, and the Y-axis shows the number of cells with that number of mC reads. **D**. Distribution of global mCG levels in each cell. The X-axis shows the global mCG level, and the Y-axis shows the number of cells with that mCG level. **E.** t-SNE embeddings of single-nucleus methylomes from control samples, including ‘HIV_pre,’ ‘Flu_pre,’ and ‘Ctrl’ groups. Colors indicate donors. **F.** t-SNE embeddings of single-nucleus methylomes from control samples, including ‘HIV_pre,’ ‘Flu_pre,’ and ‘Ctrl’ groups. Colors indicate the Leiden clusters generated based on the global mCG levels within each cell. **G.** Total number of DMRs/DMSs for each cell type (horizontal bars) as well as the number of DMRs/DMSs shared across the cell types (vertical bars). The vertical lines show the connection between the cell types that share the DMRs/DMSs.

Supplement 14 **Figure S3. Quality control and methylation changes after HIV-1 infection**. **A.** t-SNE embeddings of single-nucleus methylomes from the HIV-1 cohort, including ‘pre,’ ‘acute,’ and ‘chronic’ stages. Colors indicate the Leiden clusters generated based on the global mCG levels within each cell. **B.** t-SNE embeddings of single-nucleus methylomes from the HIV-1 cohort, including ‘pre,’ ‘acute,’ and ‘chronic’ stages. Color indicates the HIV-1 infection stages (‘pre,’ ‘acute,’ and ‘chronic’). **C.** t-SNE embeddings of single-nucleus methylomes from the HIV-1 cohort, including ‘pre,’ ‘acute,’ and ‘chronic’ stages. Color indicates the global mCG level of each cell. **D.** Proportions of naive B cells and memory B cells, as well as naive NK cells and active NK cells among the cells from the three stages (‘pre,’ ‘acute,’ and ‘chronic’). The enrichment of cells in the clusters was performed using the Chi-square test. **E.** Global mCG levels of cells in the nine cell types and three stages. **F.** Boxplot shows the CG densities at the exposure and cell type DMRs, at both the multi-CpG and single-CpG DMRs. Statistical test was done with Mann-Whitney-Wilcoxon test. **G.** Bar plots showing the count of hypo-DMRs and hyper-DMRs in each stage. **H**. Scatter plots showing the differentially methylated genes (DMGs) between each pair of stages, with the y-axis representing the p-value and the x-axis representing the difference in methylation level. **I.** Functional enrichment of the DMGs. I. Heatmap showing the normalized methylation level at the DMGs.

Supplement 15 **Figure S4. Single-cell analysis quality control and methylation changes after influenza infection. A**. t-SNE embeddings of single-nucleus methylomes from the influenza cohort, including ‘pre’ and ‘post’ stages. Colors indicate the Leiden clusters generated based on the global mCG levels within each cell. **B.** t-SNE embeddings of single-nucleus methylomes from the influenza cohort, including ‘pre’ and ‘post’ stages. Colors indicate the influenza infection stages (‘pre’ and ‘post’). **C**. t-SNE embeddings of single-nucleus methylomes from the influenza cohort, including ‘pre’ and ‘post’ stages. Colors indicate the global mCG level of each cell. **D.** Proportions of naive B cells and memory B cells, as well as naive NK cells and active NK cells among the cells from the two stages (‘pre’ and ‘post’). The enrichment of cells in the clusters was performed using the Chi-square test. **E.** Global mCG levels of cells in the nine cell types and two different stages. **F.** Bar plots showing the count of DMRs in each stage, either ‘hypo’ or ‘hyper’. **G.** Heatmap showing the genome-wide correlations between pseudo-bulk signals from each cell type in single-nucleus methylation and matched single-nucleus ATAC-seq. Red indicates a higher correlation, while blue indicates a lower correlation. 1, 2, 3 and 4 in the labels indicate different stages of influenza infection.

Supplement 16 **Figure S5. Single-cell analysis quality control and methylation changes after SARS-CoV-2 infection**. **A.** t-SNE embeddings of single-nucleus methylomes from the COVID-19 cohort, together with the healthy controls from this study. Colors indicate the Leiden clusters generated based on the global mCG levels within each cell. **B.** t-SNE embeddings of single-nucleus methylomes from the COVID-19 cohort, together with the healthy controls from this study. Colors indicate the groups of the samples (severe, moderate, and control). **C.** t-SNE embeddings of single-nucleus methylomes from the COVID-19 cohort, together with the healthy controls from this study. Colors indicate the global mCG level of each cell. **D.** Proportions of naive NK cells and active NK cells among the NK cells from the three groups (moderate, severe, and controls). The enrichment of cells in the clusters was performed using the Chi-square test. **E.** Proportions of naive B cells and memory B cells among the B cells from the three groups (moderate, severe, and controls). The enrichment of cells in the clusters was performed using the Chi-square test. **F.** Heatmap showing the column-normalized (Z-score) methylation level at the differentially methylated genes (DMGs) between two clusters of monocytes. **G.** Heatmap showing the methylation level of all samples at the differentially methylated regions (DMRs) between two clusters of monocytes. **H.** Great analysis results on the DMRs between the two monocytes; the upper panel shows the results of DMRs hypomethylated in ‘Monocyte1’, and the lower panel shows the results of DMRs hypomethylated in ‘Monocyte2’. **I.** Global mCG levels of cells in the nine cell types and three groups (moderate, severe, and controls). **J.** Bar plots showing the count of DMRs in each stage, either ‘hypo’ or ‘hyper’. **K.** Heatmap showing the DMRs identified between randomly grouped samples, which are less distinctive between groups compared to COVID-19-associated DMRs.

Supplement 17 **Figure S6. Single-cell analysis quality control and methylation changes after MRSA/MSSA infection. A**. t-SNE embeddings of single-nucleus methylomes from the MRSA/MSSA cohort, together with the healthy controls from this study. The color shows the Leiden clusters. **B.** t-SNE embeddings of single-nucleus methylomes from the MRSA/MSSA cohort, together with the healthy controls from this study. The color shows the groups of the samples (MRSA, MSSA, and controls). **C.** t-SNE embeddings of single-nucleus methylomes from the MRSA/MSSA cohort, together with the healthy controls from this study. The color shows the global mCG level of each cell. **D.** Proportions of naive B cells and memory B cells, as well as naive NK cells and active NK cells among the cells from the three groups (MRSA, MSSA, and control). The enrichment of cells in the clusters was performed using the Chi-square test. **E.** Global mCG levels of cells in the nine cell types and three different groups (MRSA, MSSA, and control). **F.** Bar plots showing the count of DMRs in each stage, either ‘hypo’ or ‘hyper’. **G.** Heatmap showing the DMRs identified between randomly grouped samples, which are less distinctive between groups compared to MRSA or MSSA-associated DMRs.

Supplement 18 **Figure S7. Single-cell analysis quality control and methylation changes in individuals working frequently or infrequently in high-risk facilities. A.** t-SNE embeddings of single-nucleus methylomes from the BA cohort and the healthy controls from this study. The color shows the Leiden clusters. **B.** t-SNE embeddings of single-nucleus methylomes from the BA cohort, together with the healthy controls from this study. The color shows the groups of the samples (frequent, infrequent, and controls). **C**. t-SNE embeddings of single-nucleus methylomes from the BA cohort, together with the healthy controls from this study. The color shows the global mCG level of each cell. **D.** Proportions of naive B cells and memory B cells, as well as naive NK cells and active NK cells among the cells from the three groups (frequent, infrequent, and control). The enrichment of cells in the clusters was performed using the Chi-square test. **E.** Global mCG levels of cells in the nine cell types and three groups (frequent, infrequent, and control). **F.** Bar plots showing the count of DMRs in each stage, either ‘hypo’ or ‘hyper’. **G.** Heatmap showing the DMRs identified between randomly grouped samples, which are less distinctive between groups compared to the frequency of working in high-risk facilities associated DMRs. *H.* Heatmap showing the correlation of methylation levels of samples from different groups at the DMRs. **I.** Heatmap showing the genome-wide correlations between pseudo-bulk signals from each cell type in single-nucleus methylation and matched single-nucleus ATAC-seq. Red indicates a higher correlation, while blue indicates a lower correlation.

Supplement 19 **Figure S8. Single-cell analysis quality control and methylation changes after OP infectio**n. **A.** t-SNE embeddings of single-nucleus methylomes from the OP cohort, together with the healthy controls from this study. The color shows the Leiden clusters. **B**. t-SNE embeddings of single-nucleus methylomes from the OP cohort, together with the healthy controls from this study. The color shows the groups of the samples (Low, Medium, High, and controls). **C**. t-SNE embeddings of single-nucleus methylomes from the OP cohort, together with the healthy controls from this study. The color shows the global mCG level of each cell. **D**. Proportions of naive B cells and memory B cells, as well as naive NK cells and active NK cells among the cells from the four groups (Low, Medium, High, and controls). The enrichment of cells in the clusters was performed using the Chi-square test. **E.** Global mCG levels of cells in the nine cell types and four groups (Low, Medium, High, and controls). **F**. Bar plots showing the count of DMRs in each stage, either ‘hypo’ or ‘hyper’. **G.** Heatmap showing the DMRs identified between randomly grouped samples, which are less distinctive between groups compared to OP-associated DMRs. **H.** Heatmap showing the genome-wide correlations between pseudo-bulk signals from each cell type in single-nucleus methylation and matched single-nucleus ATAC-seq. Red indicates a higher correlation, while blue indicates a lower correlation.

Supplement 20 **Figure S9. Hypo-methylated DMRs across exposures**. Each plot shows the total number of hypomethylated DMRs for the respective cell type (panels A-H) (horizontal bars), as well as the number of DMRs that are shared across the cell types (vertical bars). The vertical lines show the connection between the cell types that share the DMRs.

Supplement 21 **Figure S10. Hyper-methylated DMRs across exposures**. Each plot shows the total number of hypermethylated DMRs for the respective cell type (panels **A-H**) (horizontal bars), as well as the number of DMRs that are shared across the cell types (vertical bars). The vertical lines show the connection between the cell types that share the DMRs.

Supplement 22 **Figure S11. Comparison of CG context at the single-CpG and multi-CpG DMRs. A.** Boxplot shows the CG densities at the Flu associated and cell type DMRs, at both the multi-CpG and single-CpG DMRs. Statistical test was done with Mann-Whitney-Wilcoxon test. **B.** Boxplot shows the CG densities at the COVID-19 associated and cell type DMRs, at both the multi-CpG and single-CpG DMRs. Statistical test was done with Mann-Whitney-Wilcoxon test. **C.** Boxplot shows the CG densities at the MRSA/MSSA associated and cell type DMRs, at both the multi-CpG and single-CpG DMRs. Statistical test was done with Mann-Whitney-Wilcoxon test. **D.** Boxplot shows the CG densities at the high-risk facility working frequency associated and cell type DMRs, at both the multi-CpG and single-CpG DMRs. Statistical test was done with Mann-Whitney-Wilcoxon test. **E.** Boxplot shows the CG densities at the OP associated and cell type DMRs, at both the multi-CpG and single-CpG DMRs. Statistical test was done with Mann-Whitney-Wilcoxon test.

Supplement 23 **Figure S12. Motif enrichment results in all cell types and exposures.** The x-axis shows the hypo-DMRs from different groups and cell types in all the exposures, and the y-axis shows the enriched motifs. The color indicates the cell types defined by cell surface markers, and the size of the dots demonstrates the enrichment p-value.
