# Supplementary material for: Genetics and Environment Distinctively Shape the Human Immune Cell Epigenome
Source: bioRxiv. 2025 Jan 4:2023.06.29.546792. Originally published 2023 Jun 30. Preprint. [Version 2] doi: 10.1101/2023.06.29.546792 (PMC10327221; doi:10.1101/2023.06.29.546792)

# Figure S1

## A. Gating strategy

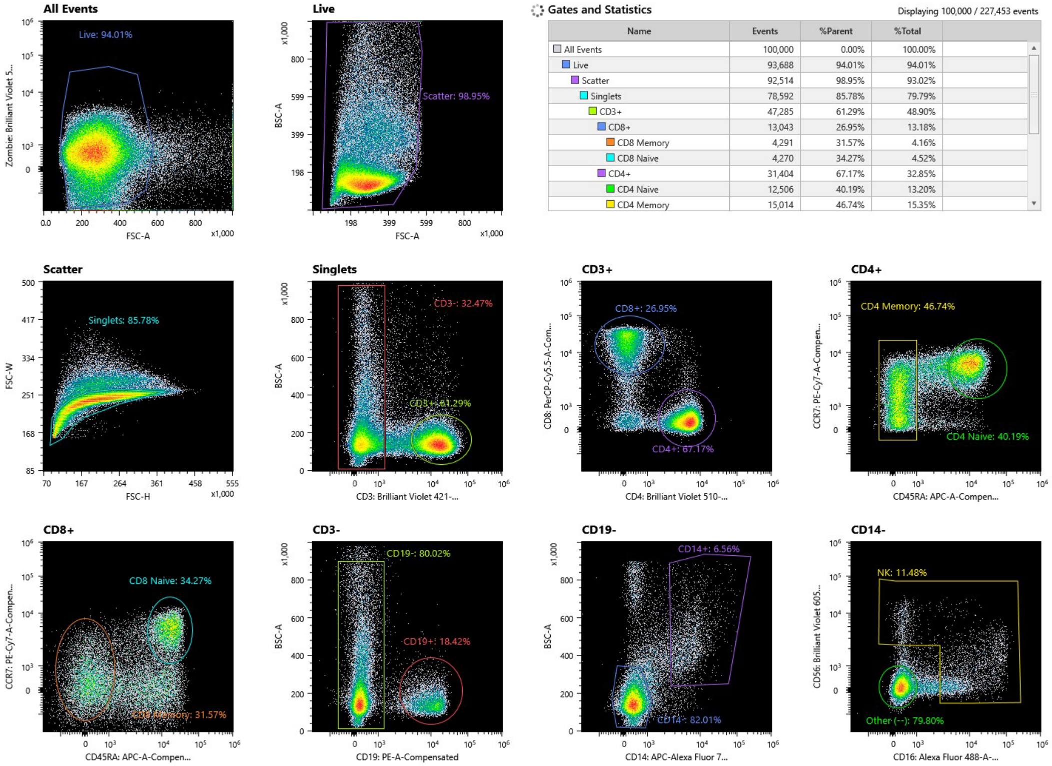


## B. Gates and Statistics from one FACS result

| Name | Events | %Parent | %Total |
|---|---|---|---|
| All Events | 100,000 | 0.00% | 100.00% |
| Live | 93,918 | 93.92% | 93.92% |
| Scatter | 92,860 | 98.87% | 92.86% |
| Singlets | 79,500 | 85.61% | 79.50% |
| CD3+ | 48,455 | 60.95% | 48.46% |
| CD8+ | 13,144 | 27.13% | 13.14% |
| CD8 Memory | 4,205 | 31.99% | 4.21% |
| CD8 Naive | 4,444 | 33.81% | 4.44% |
| CD4+ | 32,377 | 66.82% | 32.38% |
| CD4 Naive | 13,056 | 40.32% | 13.06% |
| CD4 Memory | 15,158 | 46.82% | 15.16% |
| CD3- | 25,914 | 32.60% | 25.91% |
| CD19- | 20,646 | 79.67% | 20.65% |
| CD14- | 16,736 | 81.06% | 16.74% |
| Other (--) | 13,127 | 78.44% | 13.13% |
| NK | 2,159 | 12.90% | 2.16% |
| CD14+ | 1,394 | 6.75% | 1.39% |
| CD19+ | 4,858 | 18.75% | 4.86% |

## C. Plate strategy

| Sort ID | Sort Gate | Color | Sort Mode | Cell Size | Stop Count | Timeout |
|---|---|---|---|---|---|---|
| Sort ID 1 | CD8 Memory | | Single Cell | Regular Cell | 1 | 0 |
| Sort ID 2 | NK | | Single Cell | Regular Cell | 1 | 0 |
| Sort ID 3 | CD14+ | | Single Cell | Regular Cell | 1 | 0 |
| Sort ID 4 | CD19+ | | Single Cell | Regular Cell | 1 | 0 |
| Sort ID 5 | CD4 Memory | | Single Cell | Regular Cell | 1 | 0 |
| Sort ID 6 | CD4 Naive | | Single Cell | Regular Cell | 1 | 0 |
| Sort ID 7 | CD8 Memory | | Single Cell | Regular Cell | 1 | 0 |
| Sort ID 8 | CD8 Naive | | Single Cell | Regular Cell | 1 | 0 |

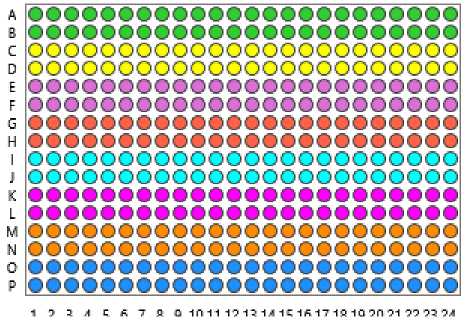

Supplement: Supplement 1 — Figure S1. FACS gating process and plate pooling strategy. A. An example gating process for one sample. B. An example of gating statistics of one sample. C. We sorted different cell types in the same plate for each sample. [file media-1.pdf]
