# Supplementary figures and images for: Genetics and Environment Distinctively Shape the Human Immune Cell Epigenome
Source: bioRxiv. 2025 Jan 4:2023.06.29.546792. Originally published 2023 Jun 30. Preprint. [Version 2] doi: 10.1101/2023.06.29.546792 (PMC10327221; doi:10.1101/2023.06.29.546792)

Figure S2

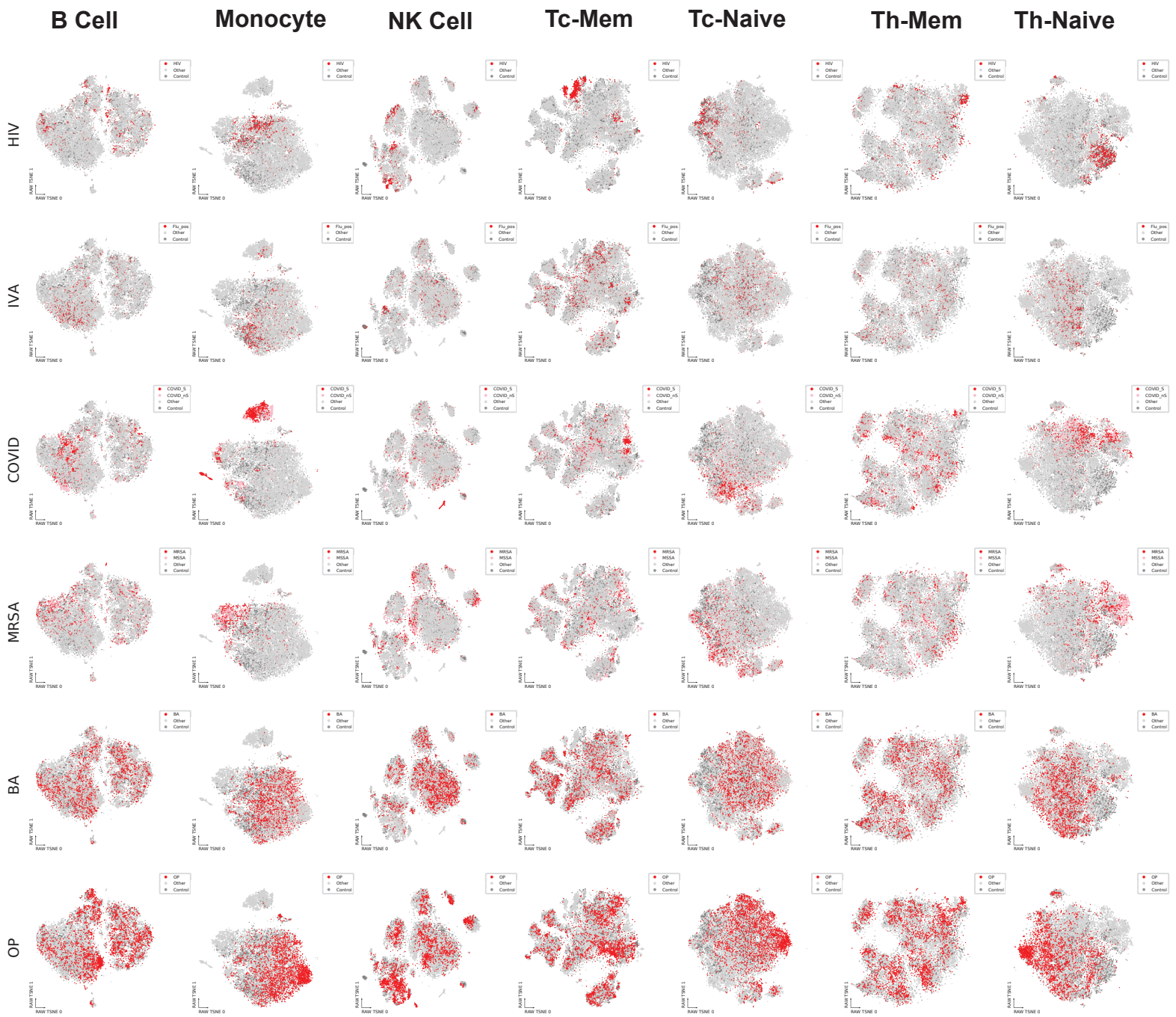

Supplement: Supplement 2 — Figure S2. Distribution of cells from each exposure within cell type UMAP. The cells from the corresponding exposure are colored in red, while other cells and control cells are colored in dark gray and gray. [file media-2.pdf]

Figure S3

A

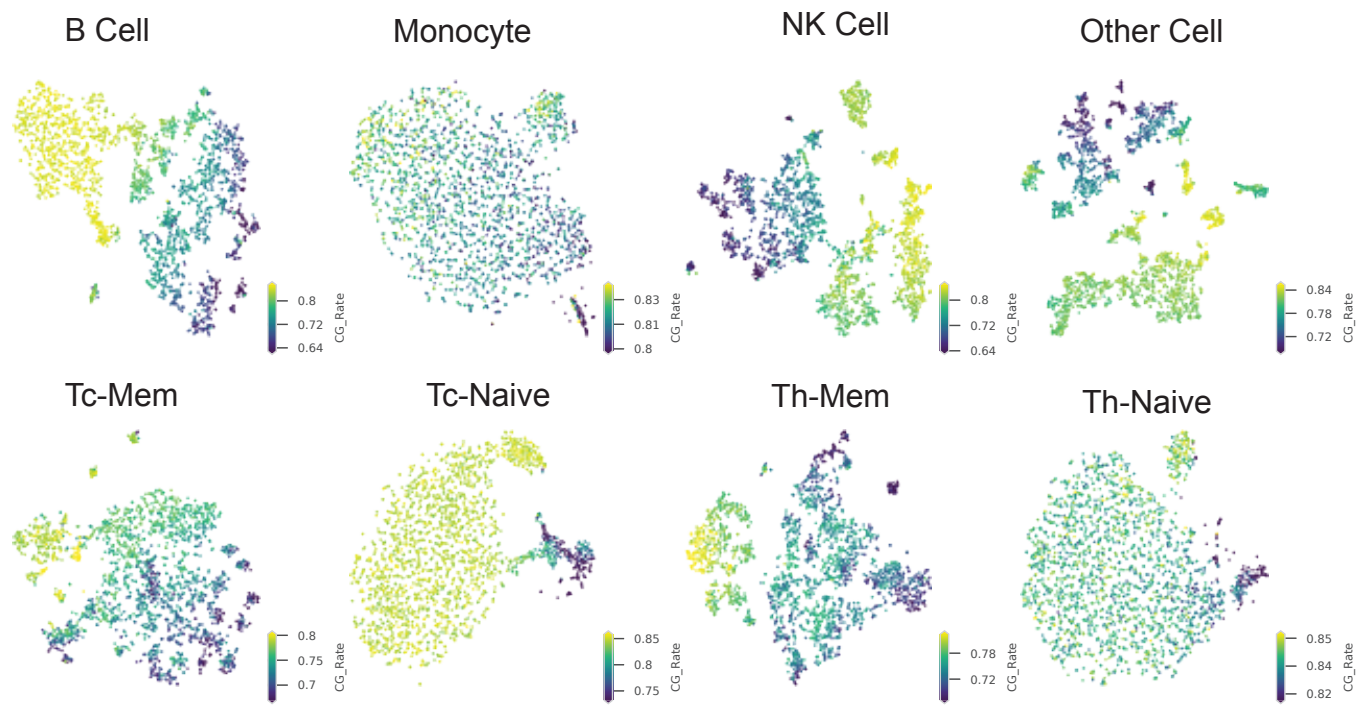

B

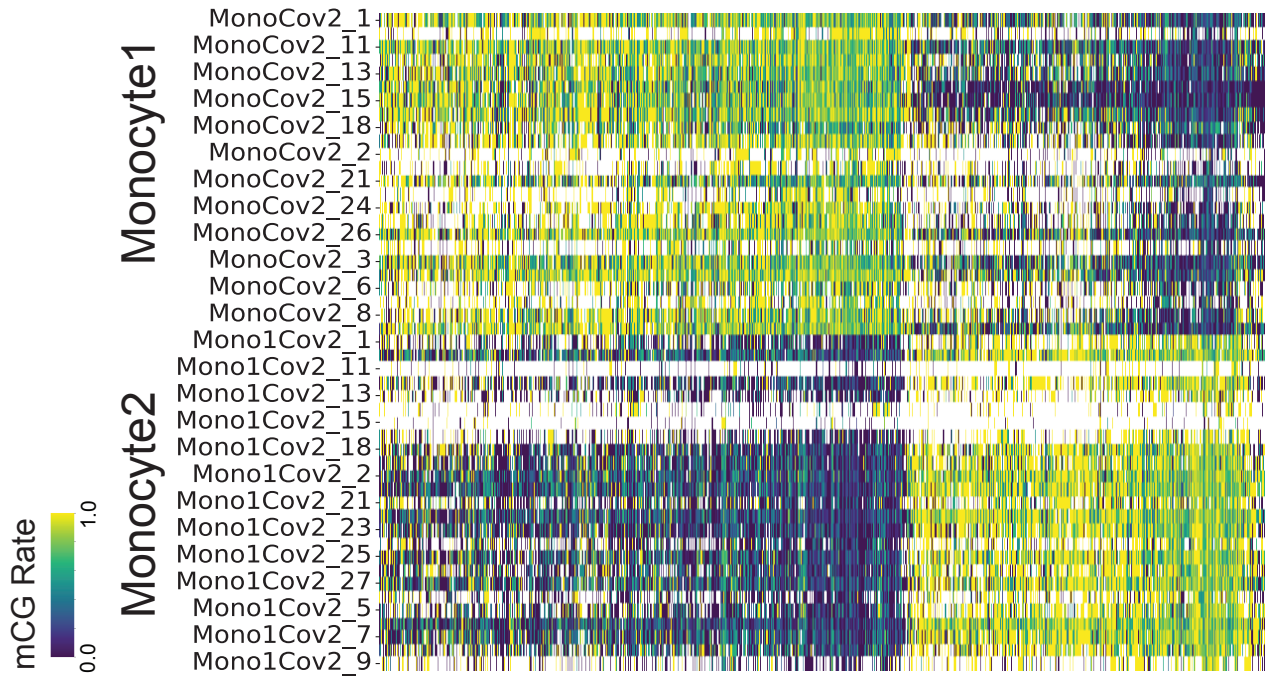

Supplement: Supplement 3 — Figure S3. Within cell type methylation difference between sub-clusters. A. The UMAP of cells from HIV exposure in each FACS cell type. The color shows the global methylation level of each cell. B. methylation level of DMGs between the two clusters of monocytes in COVID samples. [file media-3.pdf]

Figure S4

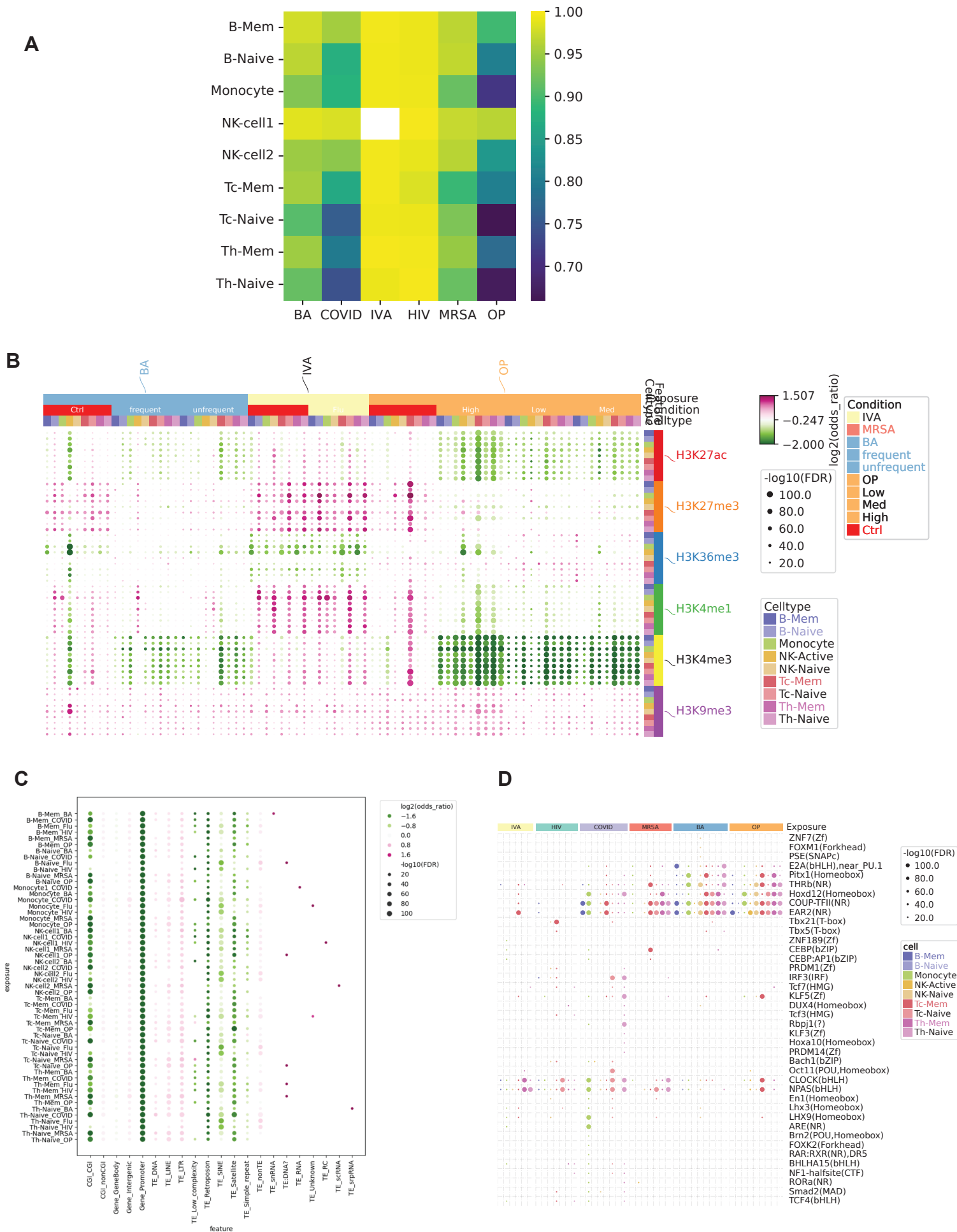

Supplement: Supplement 4 — Figure S4. Features of eDMRs. A. Heatmap shows the ratio of single CpG eDMRs in each exposure and cell type. B. Dotplot shows the enrichment of eDMRs from BA, influenza virus and OP in histone modification peaks. Each column shows the hypo-eDMRs in that condition. Color of the dots shows the enrichment or depletion in the corresponding histone modification. C. Genomic features enrichment of eDMRs in each cell type and exposure. D. Motif enrichment of hypo-eDMRs in each cell type and exposure. [file media-4.pdf]

Figure S5

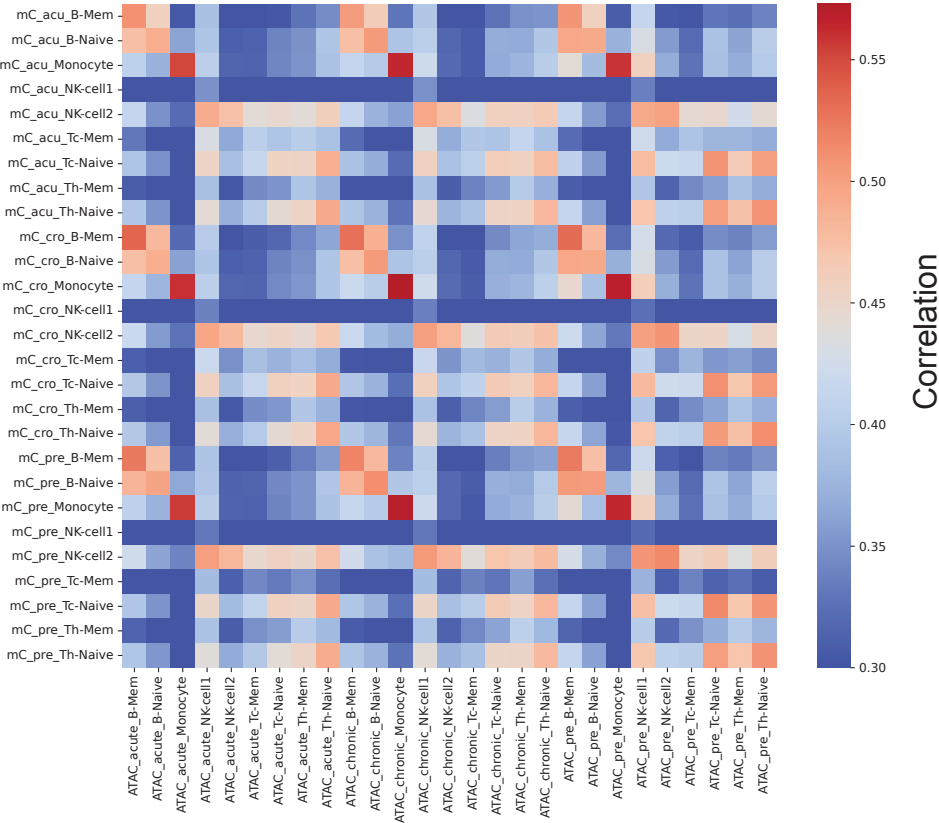

Supplement: Supplement 5 — Figure S5. Global correlation of DNA methylation and chromatin accessibility in each cell type. [file media-5.pdf]

Figure S6

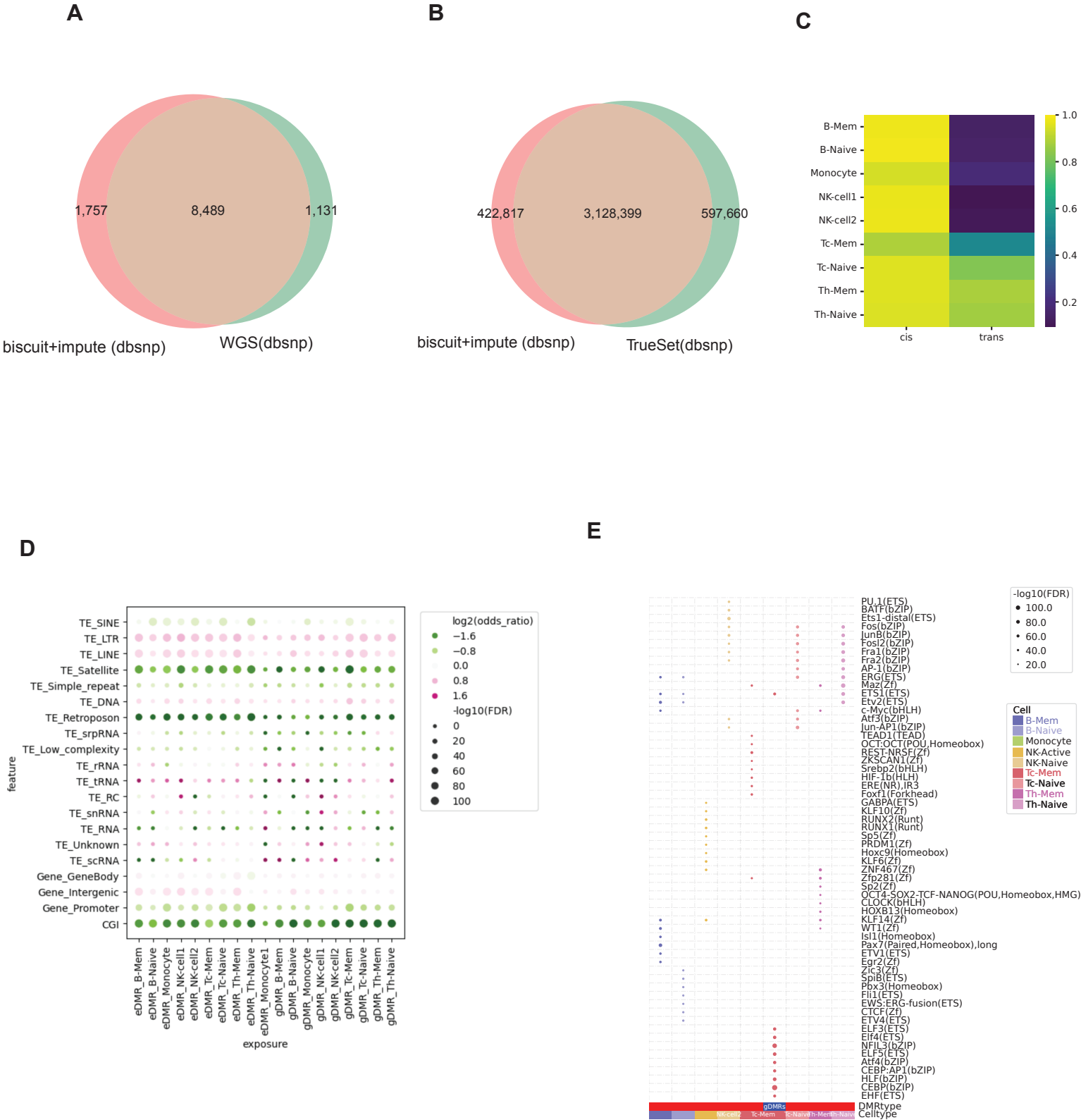

Supplement: Supplement 6 — Figure S6. Features of gDMRs. A. Venn diagram shows the overlap of SNPs called from methylation reads and WGS in a 10 Mb region. The SNPs are intersected with dbsnp. B. Venn diagram shows the overlap of SNPs called from methylation reads and ground truth SNPs for NA12878. The SNPs are intersected with dbsnp. C. Heatmap shows the ratio of single CpG gDMRs in each cell type in trans and cis. D. Genomic features enrichment of eDMRs and gDMRs. E. Motif enrichment of gDMRs and eDMRs using each other as background. [file media-6.pdf]

Figure S7

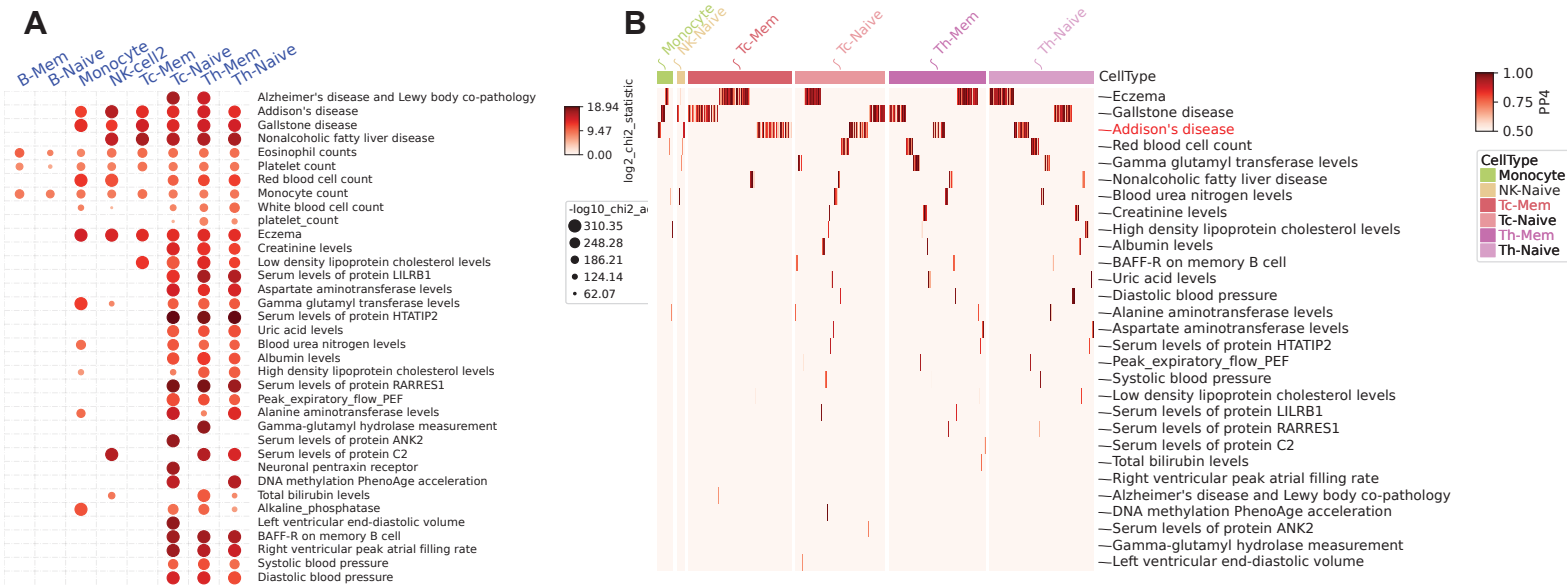

Supplement: Supplement 7 — Figure S7. Colocalization of meQTL and phenotype-associated GWAS SNPs. A. Enrichment of colocalized GWAS SNPs from each phenotype with the meQTLs from each cell type. B. Heatmap shows the distribution of colocalized meQTLs with different phenotypes in each cell type. [file media-7.pdf]
