# Supplementary material for: Human Immune Cell Epigenomic Signatures in Response to Infectious Diseases and Chemical Exposures
Source: bioRxiv. 2023 Jun 30:2023.06.29.546792. Preprint. [Version 1] doi: 10.1101/2023.06.29.546792 (PMC10327221; doi:10.1101/2023.06.29.546792)
Supplement: Supplementary file 1 [file media-12.pdf]

Figure S1

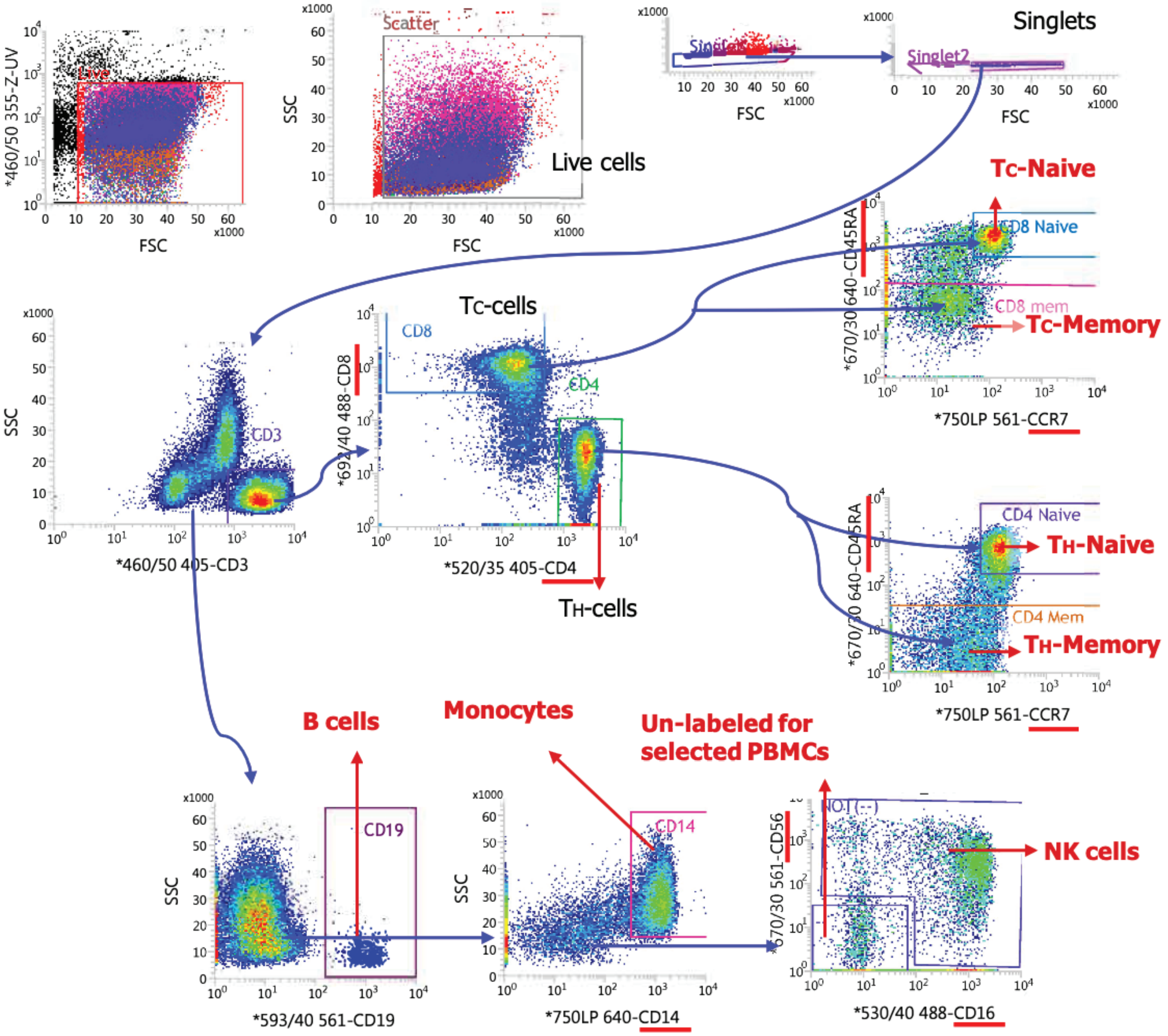

Live
*460/50 355-Z-UV
FSC
Scatter
SSC
Live cells
Singlets
Singlet2
FSC
Tc-Naive
CD8 Naive
*670/30 640-CD45RA
CD8 mem
Tc-Memory
*750LP 561-CCR7
Tc-cells
CD8
CD4
*692/40 488-CD8
*520/35 405-CD4
TH-cells
SSC
CD3
*460/50 405-CD3
CD4 Naive
TH-Naive
CD4 Mem
TH-Memory
*670/30 640-CD45RA
*750LP 561-CCR7
B cells
Monocytes
Un-labeled for selected PBMCs
CD19
SSC
*593/40 561-CD19
CD14
SSC
*750LP 640-CD14
NK cells
*670/30 561-CD56
*530/40 488-CD16
