# Supplementary material for: Human Immune Cell Epigenomic Signatures in Response to Infectious Diseases and Chemical Exposures
Source: bioRxiv. 2023 Jun 30:2023.06.29.546792. Preprint. [Version 1] doi: 10.1101/2023.06.29.546792 (PMC10327221; doi:10.1101/2023.06.29.546792)
Supplement: Supplementary file 2 [file media-13.pdf]

Figure S2

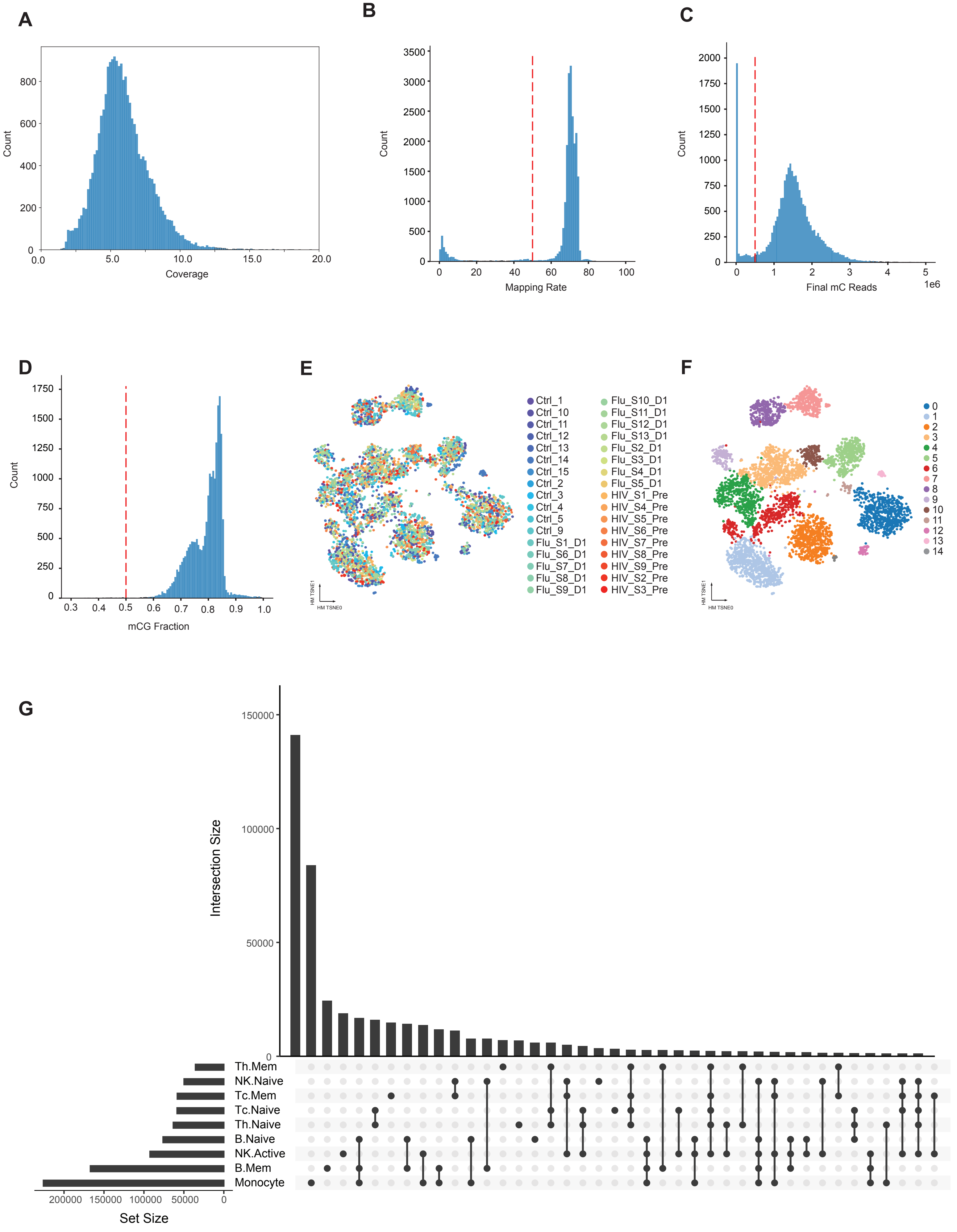
A
Count
Coverage
B
Count
Mapping Rate
C
Count
Final mC Reads
1e6
D
Count
mCG Fraction
E
Ctrl_1
Ctrl_10
Ctrl_11
Ctrl_12
Ctrl_13
Ctrl_14
Ctrl_15
Ctrl_2
Ctrl_3
Ctrl_4
Ctrl_5
Ctrl_9
Flu_S1_D1
Flu_S6_D1
Flu_S7_D1
Flu_S8_D1
Flu_S9_D1
Flu_S10_D1
Flu_S11_D1
Flu_S12_D1
Flu_S13_D1
Flu_S2_D1
Flu_S3_D1
Flu_S4_D1
Flu_S5_D1
HIV_S1_Pre
HIV_S4_Pre
HIV_S5_Pre
HIV_S6_Pre
HIV_S7_Pre
HIV_S8_Pre
HIV_S9_Pre
HIV_S2_Pre
HIV_S3_Pre
HM TSNE1
HM TSNE0
F
G
Intersection Size
Th.Mem
NK.Naive
Tc.Mem
Tc.Naive
Th.Naive
B.Naive
NK.Active
B.Mem
Monocyte
Set Size
