# Supplementary material for: Human Immune Cell Epigenomic Signatures in Response to Infectious Diseases and Chemical Exposures
Source: bioRxiv. 2023 Jun 30:2023.06.29.546792. Preprint. [Version 1] doi: 10.1101/2023.06.29.546792 (PMC10327221; doi:10.1101/2023.06.29.546792)
Supplement: Supplementary file 3 [file media-14.pdf]

Figure S3

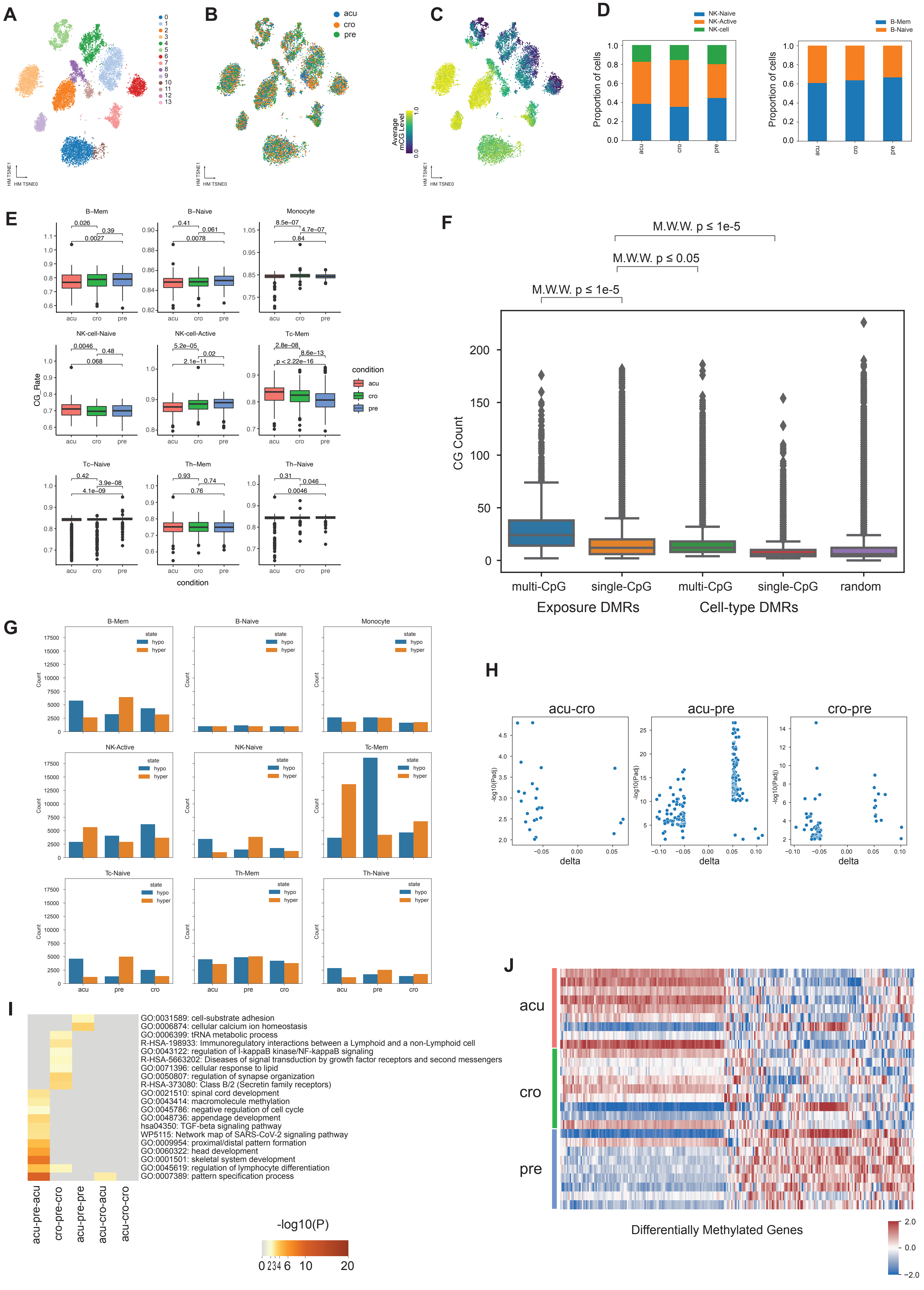

A
B
acu
cro
pre
C
Average mCG Level
D
NK-Naive
NK-Active
NK-cell
B-Mem
B-Naive
Proportion of cells
E
B–Mem
B–Naive
Monocyte
NK-cell-Naive
NK-cell-Active
Tc-Mem
Tc–Naive
Th–Mem
Th–Naive
CG_Rate
condition
F
M.W.W. p ≤ 1e-5
M.W.W. p ≤ 0.05
M.W.W. p ≤ 1e-5
CG Count
multi-CpG
single-CpG
multi-CpG
single-CpG
random
Exposure DMRs
Cell-type DMRs
G
state
hypo
hyper
Count
H
acu-cro
acu-pre
cro-pre
-log10(Padj)
delta
I
GO:0031589: cell-substrate adhesion
GO:0006874: cellular calcium ion homeostasis
GO:0006399: tRNA metabolic process
R-HSA-198933: Immunoregulatory interactions between a Lymphoid and a non-Lymphoid cell
GO:0043122: regulation of I-kappaB kinase/NF-kappaB signaling
R-HSA-5663202: Diseases of signal transduction by growth factor receptors and second messengers
GO:0071396: cellular response to lipid
GO:0050807: regulation of synapse organization
R-HSA-373080: Class B/2 (Secretin family receptors)
GO:0021510: spinal cord development
GO:0043414: macromolecule methylation
GO:0045786: negative regulation of cell cycle
GO:0048736: appendage development
hsa04350: TGF-beta signaling pathway
WP5115: Network map of SARS-CoV-2 signaling pathway
GO:0009954: proximal/distal pattern formation
GO:0060322: head development
GO:0001501: skeletal system development
GO:0045619: regulation of lymphocyte differentiation
GO:0007389: pattern specification process
acu-pre-acu
cro-pre-cro
acu-pre-pre
acu-cro-acu
acu-cro-cro
-log10(P)
J
acu
cro
pre
Differentially Methylated Genes
