# Supplementary material for: Human Immune Cell Epigenomic Signatures in Response to Infectious Diseases and Chemical Exposures
Source: bioRxiv. 2023 Jun 30:2023.06.29.546792. Preprint. [Version 1] doi: 10.1101/2023.06.29.546792 (PMC10327221; doi:10.1101/2023.06.29.546792)
Supplement: Supplementary file 4 [file media-15.pdf]

Figure S4

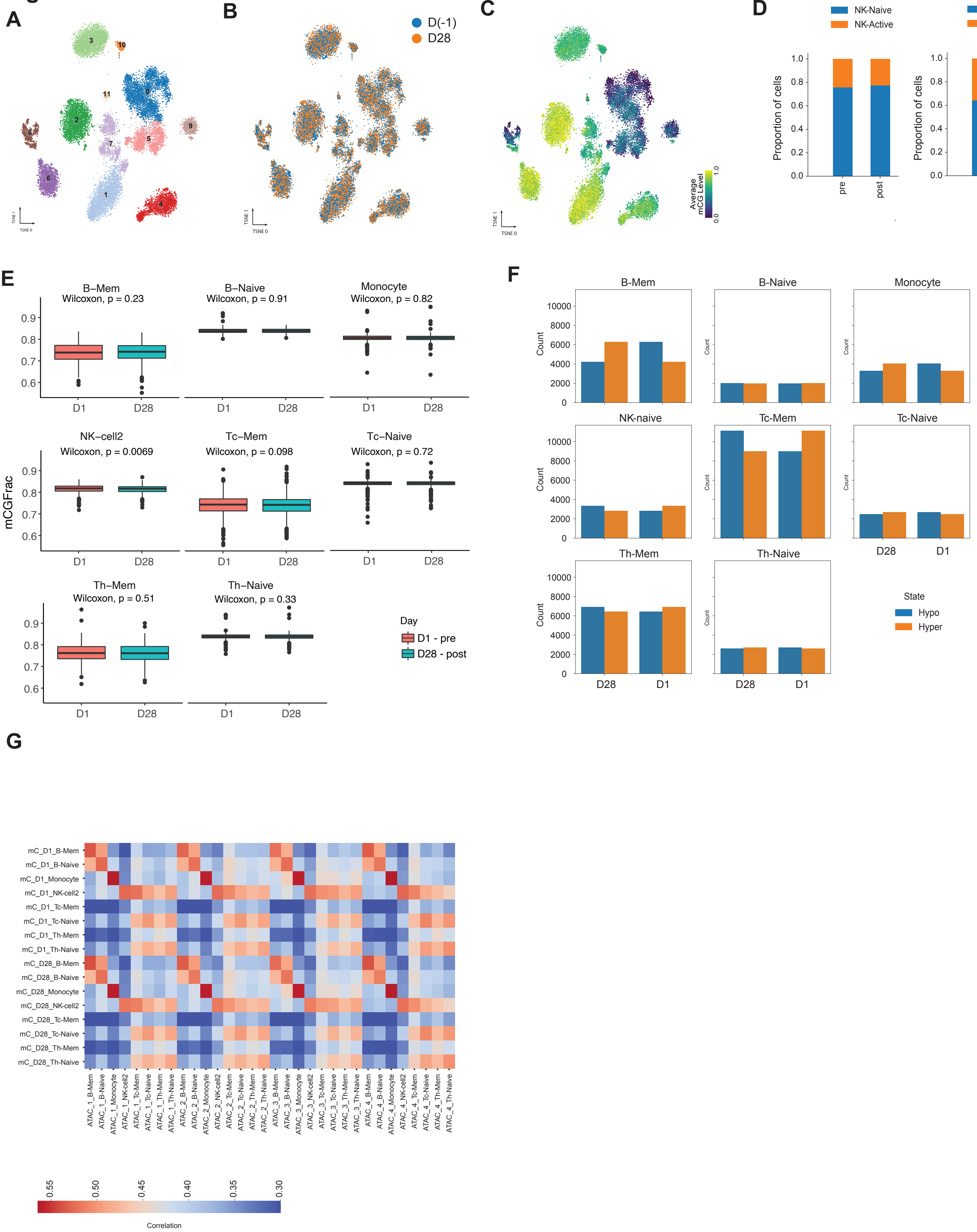

A
B
D(-1)
D28
C
Average mCG Level
D
NK-Naive
NK-Active
B-Naive
B-Mem
Proportion of cells
pre
post
E
B−Mem
Wilcoxon, p = 0.23
B−Naive
Wilcoxon, p = 0.91
Monocyte
Wilcoxon, p = 0.82
NK−cell2
Wilcoxon, p = 0.0069
Tc−Mem
Wilcoxon, p = 0.098
Tc−Naive
Wilcoxon, p = 0.72
Th−Mem
Wilcoxon, p = 0.51
Th−Naive
Wilcoxon, p = 0.33
mCGFrac
Day
D1 - pre
D28 - post
F
B-Mem
B-Naive
Monocyte
NK-naive
Tc-Mem
Tc-Naive
Th-Mem
Th-Naive
Count
State
Hypo
Hyper
G
Correlation
