# Supplementary material for: Human Immune Cell Epigenomic Signatures in Response to Infectious Diseases and Chemical Exposures
Source: bioRxiv. 2023 Jun 30:2023.06.29.546792. Preprint. [Version 1] doi: 10.1101/2023.06.29.546792 (PMC10327221; doi:10.1101/2023.06.29.546792)
Supplement: Supplementary file 5 [file media-16.pdf]

Figure S5

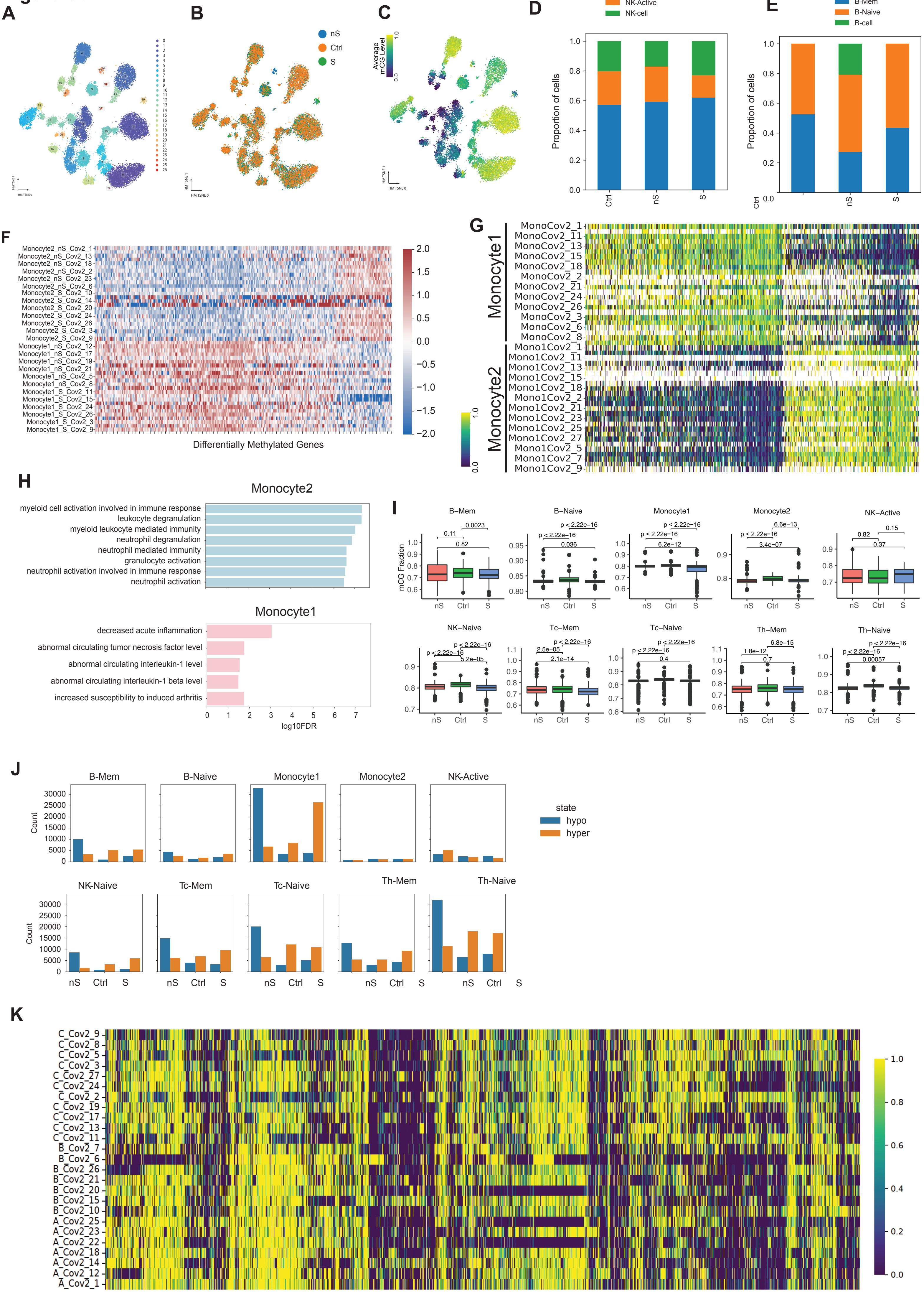
A
B
nS
Ctrl
S
C
Average mCG Level
D
NK-Naive
NK-Active
NK-cell
Proportion of cells
E
B-Mem
B-Naive
B-cell
F
Differentially Methylated Genes
G
Monocyte1
Monocyte2
H
Monocyte2
myeloid cell activation involved in immune response
leukocyte degranulation
myeloid leukocyte mediated immunity
neutrophil degranulation
neutrophil mediated immunity
granulocyte activation
neutrophil activation involved in immune response
neutrophil activation
Monocyte1
decreased acute inflammation
abnormal circulating tumor necrosis factor level
abnormal circulating interleukin-1 level
abnormal circulating interleukin-1 beta level
increased susceptibility to induced arthritis
log10FDR
I
mCG Fraction
B–Mem
B–Naive
Monocyte1
Monocyte2
NK–Active
NK–Naive
Tc–Mem
Tc–Naive
Th–Mem
Th–Naive
J
B-Mem
B-Naive
Monocyte1
Monocyte2
NK-Active
NK-Naive
Tc-Mem
Tc-Naive
Th-Mem
Th-Naive
Count
state
hypo
hyper
K
