# Supplementary material for: Human Immune Cell Epigenomic Signatures in Response to Infectious Diseases and Chemical Exposures
Source: bioRxiv. 2023 Jun 30:2023.06.29.546792. Preprint. [Version 1] doi: 10.1101/2023.06.29.546792 (PMC10327221; doi:10.1101/2023.06.29.546792)
Supplement: Supplementary file 6 [file media-17.pdf]

Figure S6

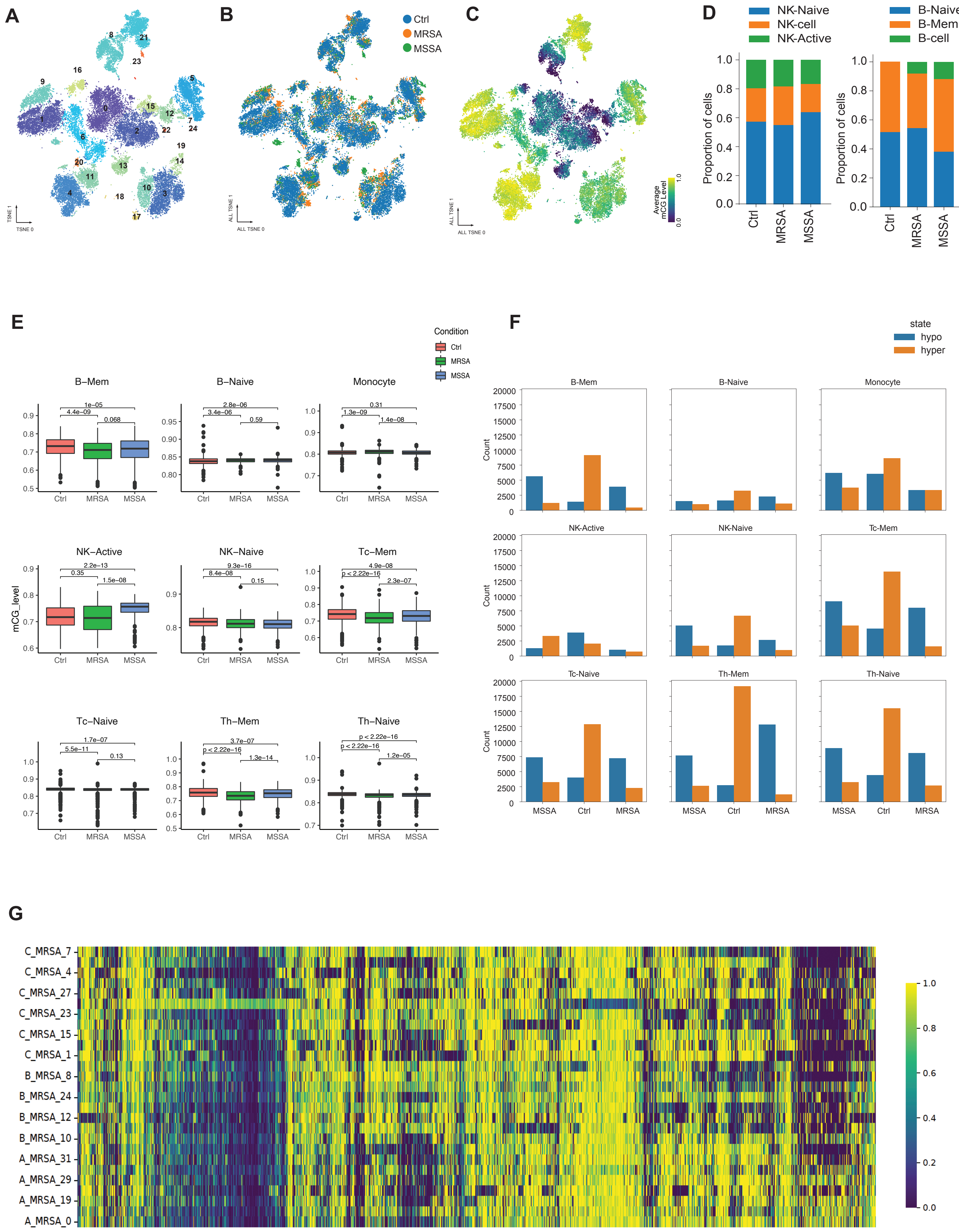
A
B
Ctrl
MRSA
MSSA
C
Average mCG Level
D
NK-Naive
NK-cell
NK-Active
B-Naive
B-Mem
B-cell
Proportion of cells
E
Condition
B–Mem
B–Naive
Monocyte
NK–Active
NK–Naive
Tc–Mem
Tc–Naive
Th–Mem
Th–Naive
mCG_level
F
state
hypo
hyper
Count
G
C_MRSA_7
C_MRSA_4
C_MRSA_27
C_MRSA_23
C_MRSA_15
C_MRSA_1
B_MRSA_8
B_MRSA_24
B_MRSA_12
B_MRSA_10
A_MRSA_31
A_MRSA_29
A_MRSA_19
A_MRSA_0
