# Supplementary material for: Human Immune Cell Epigenomic Signatures in Response to Infectious Diseases and Chemical Exposures
Source: bioRxiv. 2023 Jun 30:2023.06.29.546792. Preprint. [Version 1] doi: 10.1101/2023.06.29.546792 (PMC10327221; doi:10.1101/2023.06.29.546792)
Supplement: Supplementary file 8 [file media-19.pdf]

Figure S8

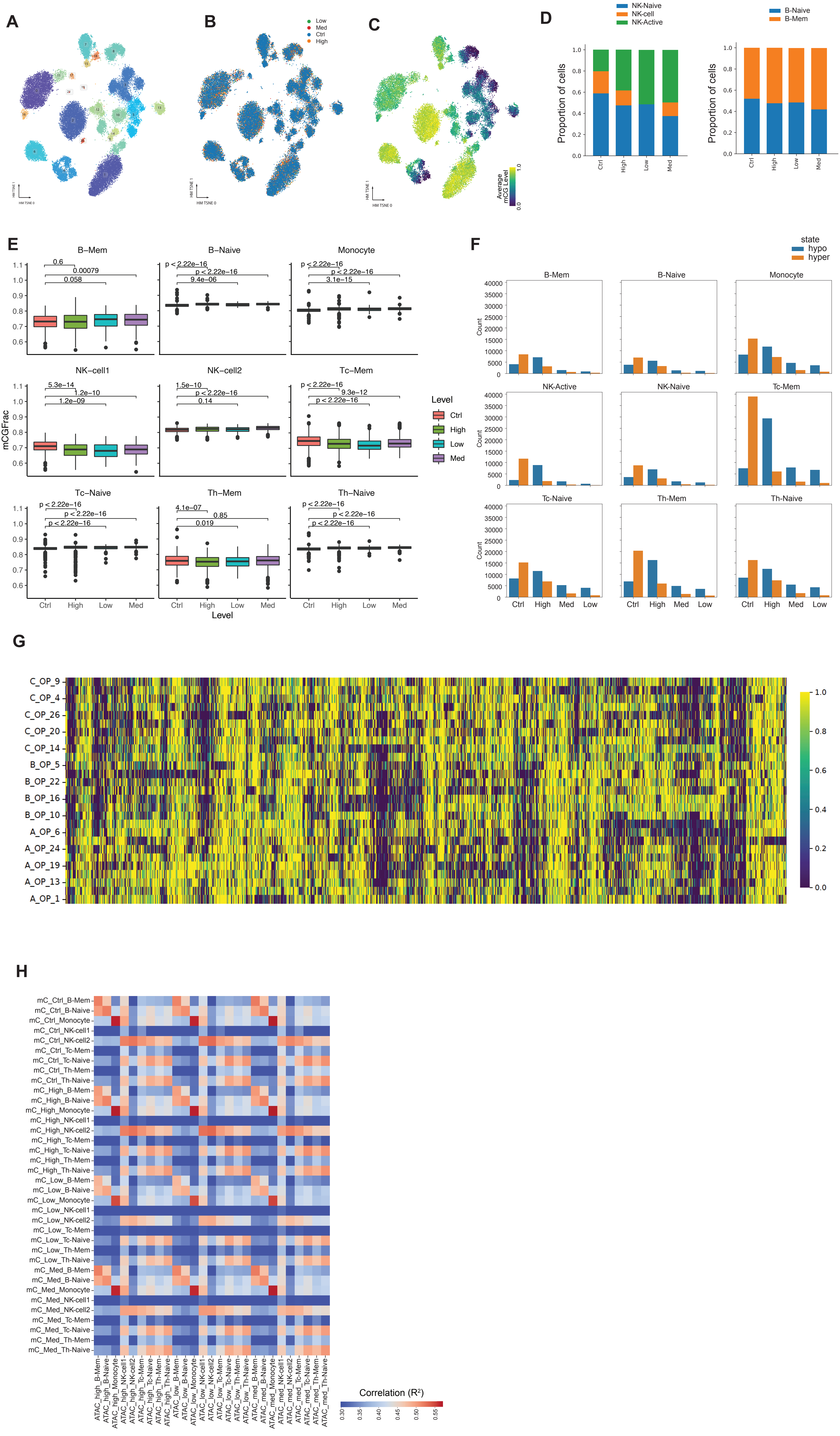

A
B
Low
Med
Ctrl
High
C
Average mCG Level
D
NK-Naive
NK-cell
NK-Active
B-Naive
B-Mem
Proportion of cells
Ctrl
High
Low
Med
E
B–Mem
B–Naive
Monocyte
NK–cell1
NK–cell2
Tc–Mem
Tc–Naive
Th–Mem
Th–Naive
mCGFrac
Level
F
state
hypo
hyper
B-Mem
B-Naive
Monocyte
NK-Active
NK-Naive
Tc-Mem
Tc-Naive
Th-Mem
Th-Naive
Count
G
C_OP_9
C_OP_4
C_OP_26
C_OP_20
C_OP_14
B_OP_5
B_OP_22
B_OP_16
B_OP_10
A_OP_6
A_OP_24
A_OP_19
A_OP_13
A_OP_1
H
Correlation (R²)
