# Supplementary material for: Human Immune Cell Epigenomic Signatures in Response to Infectious Diseases and Chemical Exposures
Source: bioRxiv. 2023 Jun 30:2023.06.29.546792. Preprint. [Version 1] doi: 10.1101/2023.06.29.546792 (PMC10327221; doi:10.1101/2023.06.29.546792)
Supplement: Supplementary file 9 [file media-20.pdf]

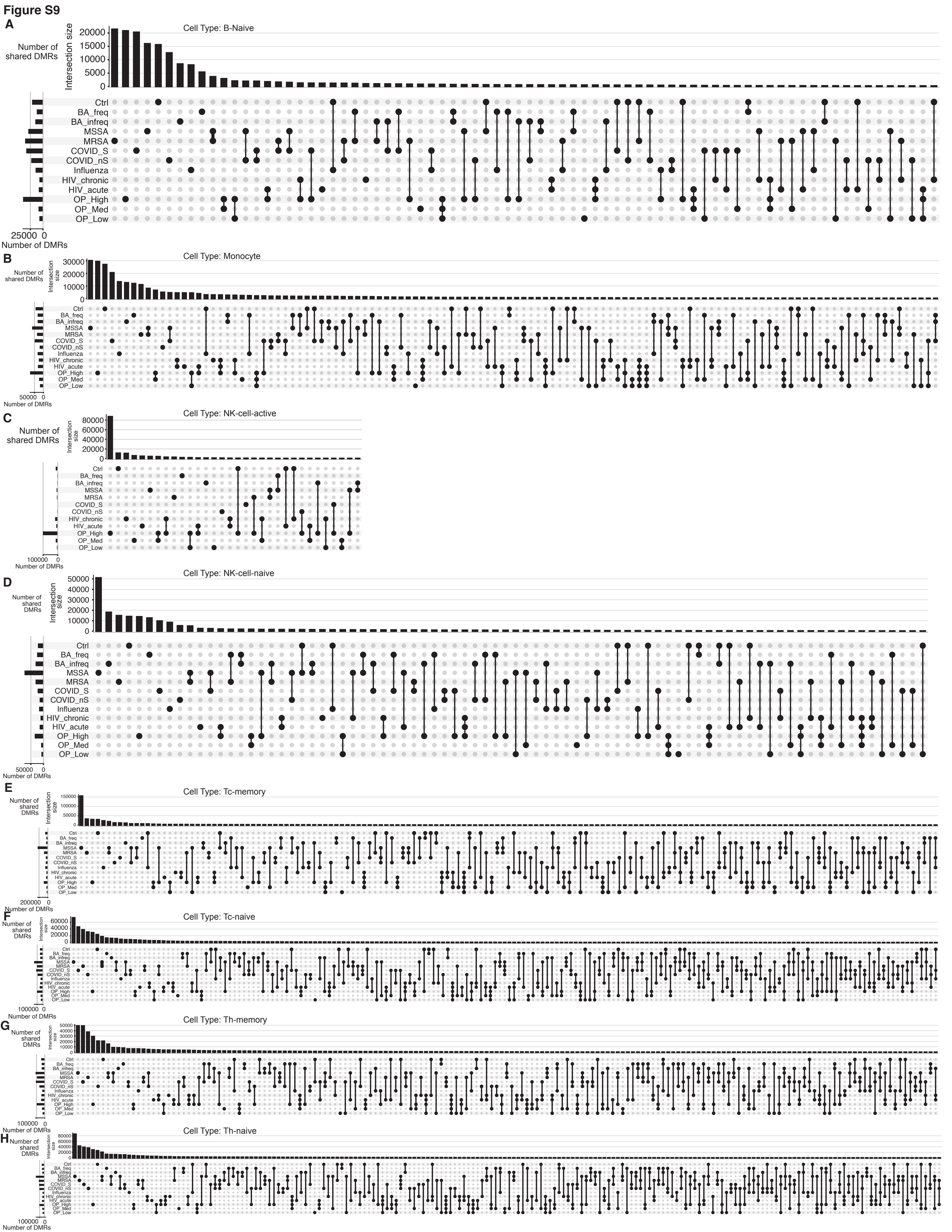
Figure S9
A
Cell Type: B-Naive
Number of shared DMRs
Intersection size
Ctrl
BA_freq
BA_infreq
MSSA
MRSA
COVID_S
COVID_nS
Influenza
HIV_chronic
HIV_acute
OP_High
OP_Med
OP_Low
Number of DMRs
B
Cell Type: Monocyte
C
Cell Type: NK-cell-active
D
Cell Type: NK-cell-naive
E
Cell Type: Tc-memory
F
Cell Type: Tc-naive
G
Cell Type: Th-memory
H
Cell Type: Th-naive
