# Supplementary material for: Human Immune Cell Epigenomic Signatures in Response to Infectious Diseases and Chemical Exposures
Source: bioRxiv. 2023 Jun 30:2023.06.29.546792. Preprint. [Version 1] doi: 10.1101/2023.06.29.546792 (PMC10327221; doi:10.1101/2023.06.29.546792)
Supplement: Supplementary file 10 [file media-21.pdf]

Figure S10

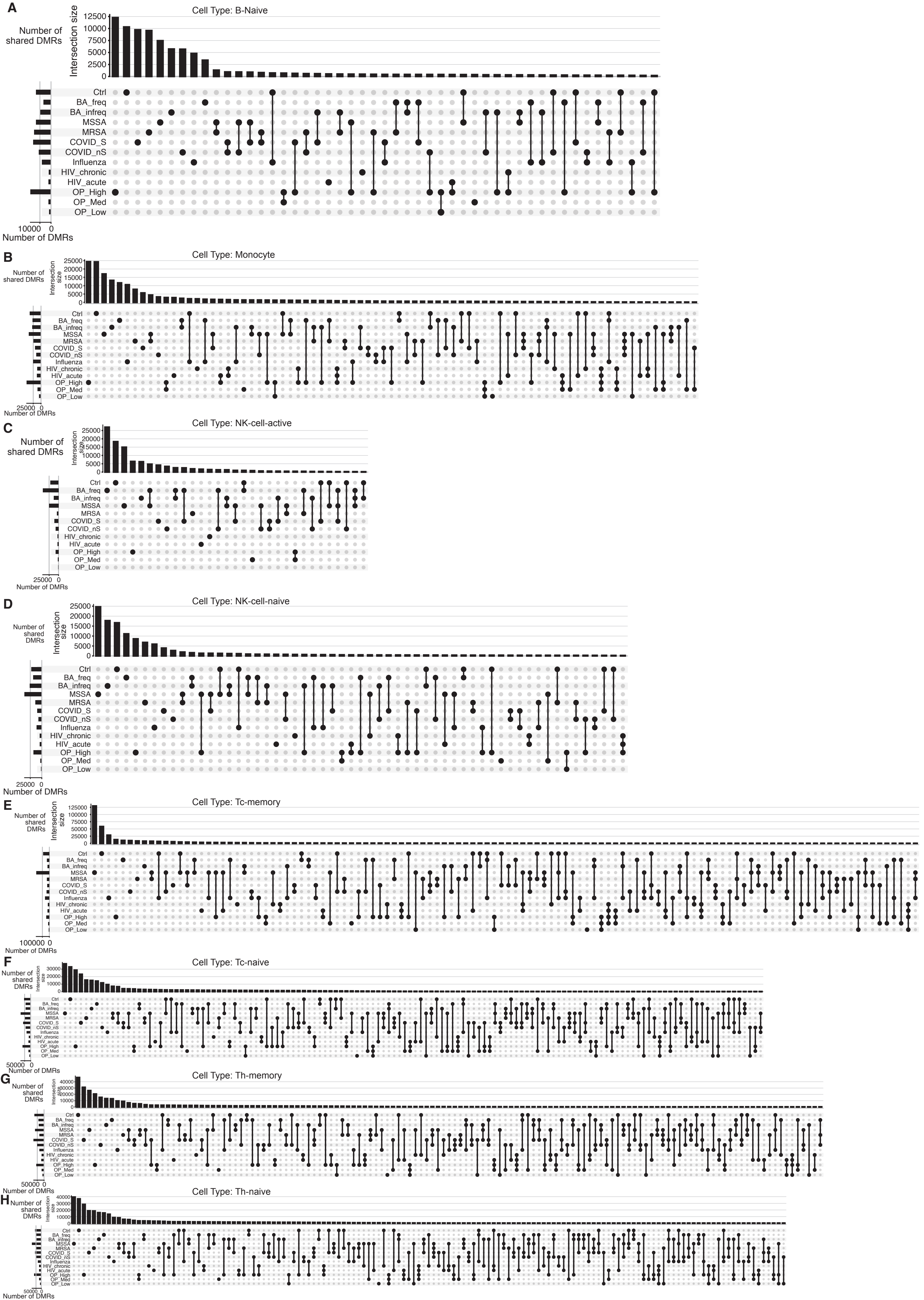
A
Cell Type: B-Naive
Number of shared DMRs
Intersection size
12500
10000
7500
5000
2500
0
Ctrl
BA_freq
BA_infreq
MSSA
MRSA
COVID_S
COVID_nS
Influenza
HIV_chronic
HIV_acute
OP_High
OP_Med
OP_Low
10000 0
Number of DMRs
B
Cell Type: Monocyte
Number of shared DMRs
Intersection size
25000
20000
15000
10000
5000
0
25000 0
Number of DMRs
C
Cell Type: NK-cell-active
Number of shared DMRs
Intersection size
25000
20000
15000
10000
5000
0
25000 0
Number of DMRs
D
Cell Type: NK-cell-naive
Number of shared DMRs
Intersection size
25000
20000
15000
10000
5000
0
25000 0
Number of DMRs
E
Cell Type: Tc-memory
Number of shared DMRs
Intersection size
125000
100000
75000
50000
25000
0
100000 0
Number of DMRs
F
Cell Type: Tc-naive
Number of shared DMRs
Intersection size
30000
20000
10000
0
50000 0
Number of DMRs
G
Cell Type: Th-memory
Number of shared DMRs
Intersection size
40000
30000
20000
10000
0
50000 0
Number of DMRs
H
Cell Type: Th-naive
Number of shared DMRs
Intersection size
40000
30000
20000
10000
0
50000 0
Number of DMRs
