# Supplementary material for: Human Immune Cell Epigenomic Signatures in Response to Infectious Diseases and Chemical Exposures
Source: bioRxiv. 2023 Jun 30:2023.06.29.546792. Preprint. [Version 1] doi: 10.1101/2023.06.29.546792 (PMC10327221; doi:10.1101/2023.06.29.546792)
Supplement: Supplementary file 11 [file media-22.pdf]

Figure S11

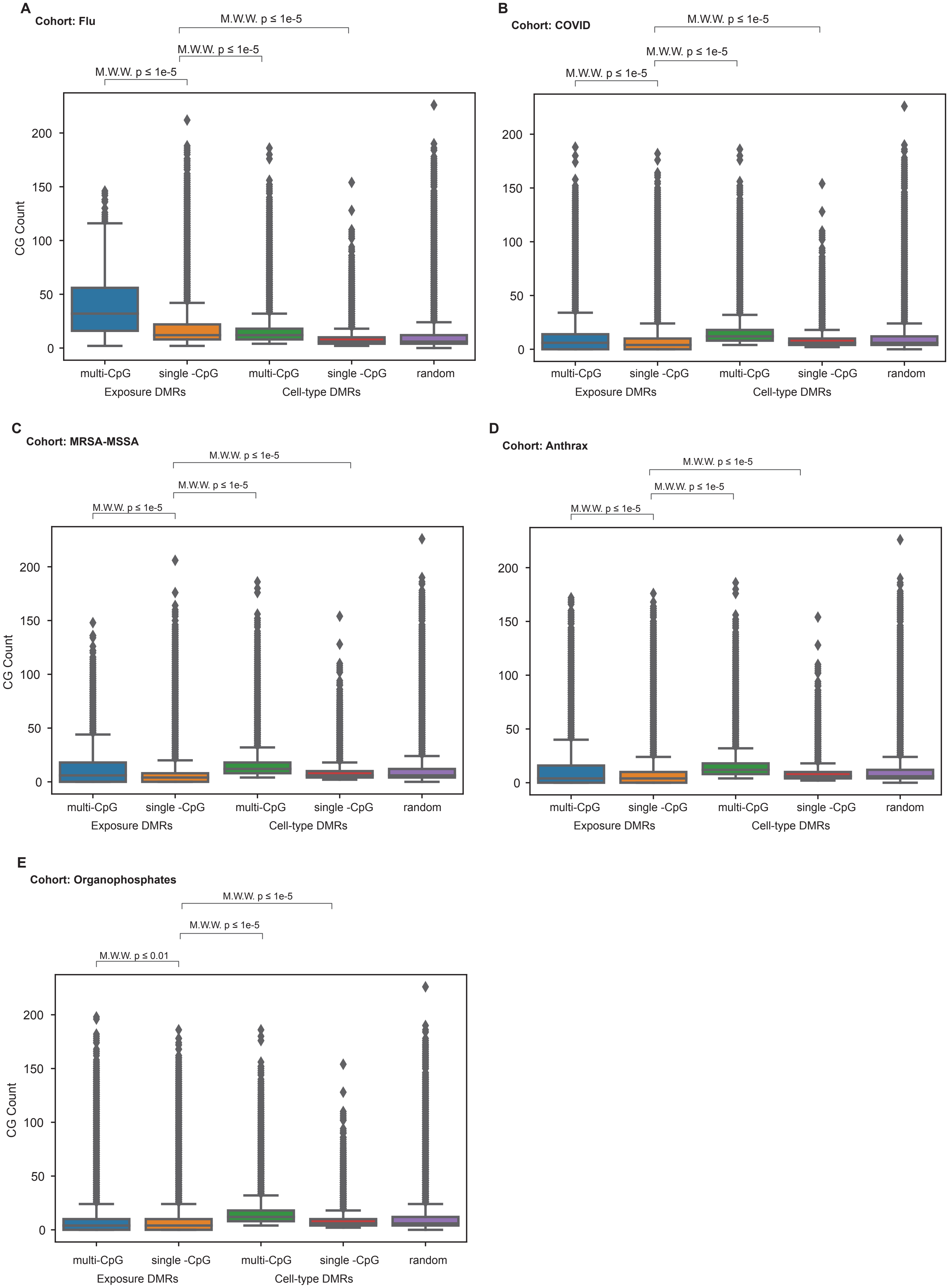

A
Cohort: Flu
M.W.W. p ≤ 1e-5
M.W.W. p ≤ 1e-5
M.W.W. p ≤ 1e-5
CG Count
0
50
100
150
200
multi-CpG
single -CpG
multi-CpG
single -CpG
random
Exposure DMRs
Cell-type DMRs
B
Cohort: COVID
M.W.W. p ≤ 1e-5
M.W.W. p ≤ 1e-5
M.W.W. p ≤ 1e-5
0
50
100
150
200
multi-CpG
single -CpG
multi-CpG
single -CpG
random
Exposure DMRs
Cell-type DMRs
C
Cohort: MRSA-MSSA
M.W.W. p ≤ 1e-5
M.W.W. p ≤ 1e-5
M.W.W. p ≤ 1e-5
CG Count
0
50
100
150
200
multi-CpG
single -CpG
multi-CpG
single -CpG
random
Exposure DMRs
Cell-type DMRs
D
Cohort: Anthrax
M.W.W. p ≤ 1e-5
M.W.W. p ≤ 1e-5
M.W.W. p ≤ 1e-5
0
50
100
150
200
multi-CpG
single -CpG
multi-CpG
single -CpG
random
Exposure DMRs
Cell-type DMRs
E
Cohort: Organophosphates
M.W.W. p ≤ 1e-5
M.W.W. p ≤ 1e-5
M.W.W. p ≤ 0.01
CG Count
0
50
100
150
200
multi-CpG
single -CpG
multi-CpG
single -CpG
random
Exposure DMRs
Cell-type DMRs
