# Supplementary material for: Human Immune Cell Epigenomic Signatures in Response to Infectious Diseases and Chemical Exposures
Source: bioRxiv. 2023 Jun 30:2023.06.29.546792. Preprint. [Version 1] doi: 10.1101/2023.06.29.546792 (PMC10327221; doi:10.1101/2023.06.29.546792)
Supplement: Supplementary file 12 [file media-23.pdf]

Figure S12

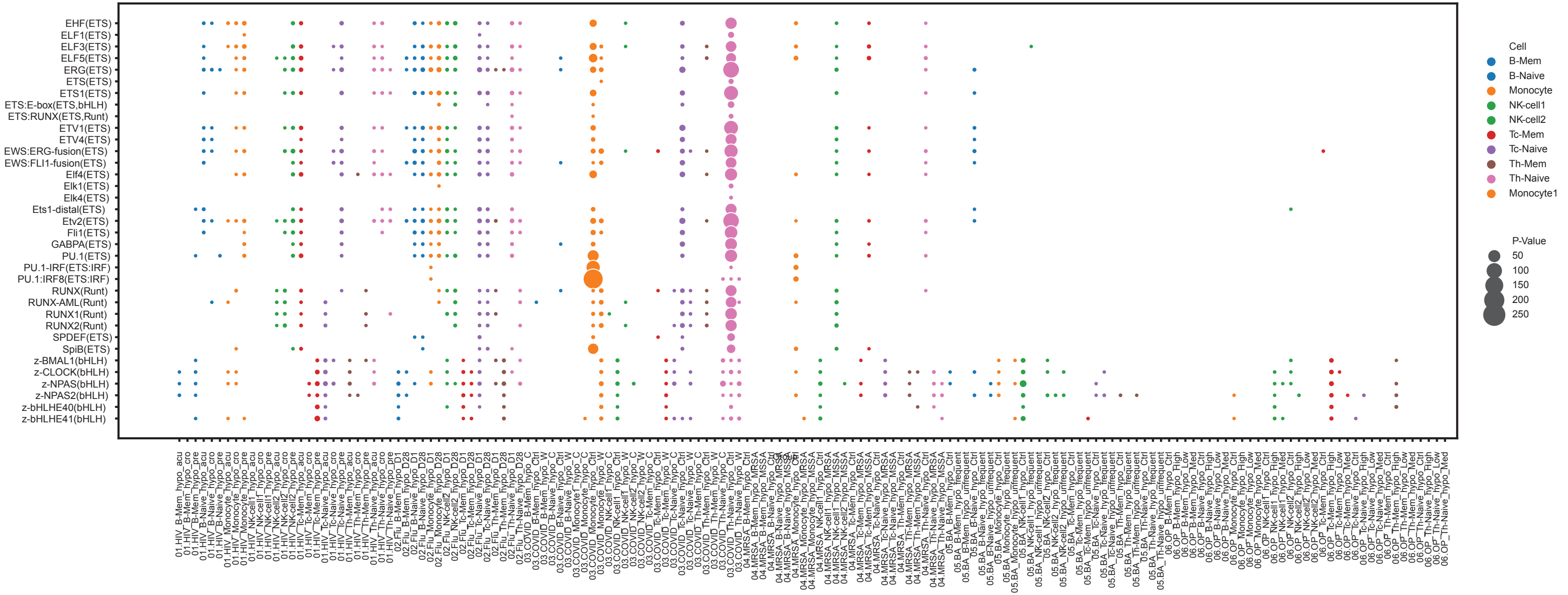

EHF(ETS)
ELF1(ETS)
ELF3(ETS)
ELF5(ETS)
ERG(ETS)
ETS(ETS)
ETS1(ETS)
ETS:E-box(ETS,bHLH)
ETS:RUNX(ETS,Runt)
ETV1(ETS)
ETV4(ETS)
EWS:ERG-fusion(ETS)
EWS:FLI1-fusion(ETS)
Elf4(ETS)
Elk1(ETS)
Elk4(ETS)
Ets1-distal(ETS)
Etv2(ETS)
Fli1(ETS)
GABPA(ETS)
PU.1(ETS)
PU.1-IRF(ETS:IRF)
PU.1:IRF8(ETS:IRF)
RUNX(Runt)
RUNX-AML(Runt)
RUNX1(Runt)
RUNX2(Runt)
SPDEF(ETS)
SpiB(ETS)
z-BMAL1(bHLH)
z-CLOCK(bHLH)
z-NPAS(bHLH)
z-NPAS2(bHLH)
z-bHLHE40(bHLH)
z-bHLHE41(bHLH)
Cell
B-Mem
B-Naive
Monocyte
NK-cell1
NK-cell2
Tc-Mem
Tc-Naive
Th-Mem
Th-Naive
Monocyte1
P-Value
50
100
150
200
250
